# The macrocyclic lactone oxacyclododecindione reduces fibrosis progression

**DOI:** 10.3389/fphar.2023.1200164

**Published:** 2023-06-13

**Authors:** Sabrina Saurin, Myriam Meineck, Markus Rohr, Wilfried Roth, Till Opatz, Gerhard Erkel, Andrea Pautz, Julia Weinmann-Menke

**Affiliations:** ^1^ Department of Nephrology, Center of Immunotherapy, Medical Center of the Johannes-Gutenberg University Mainz, Mainz, Germany; ^2^ Department of Molecular Biotechnology and Systems Biology, RPTU Kaiserslautern-Landau, Kaiserslautern, Germany; ^3^ Institute of Pathology, Medical Center of the Johannes-Gutenberg University Mainz, Mainz, Germany; ^4^ Department of Chemistry, Johannes-Gutenberg University Mainz, Mainz, Germany; ^5^ Institute of Pharmacology, Medical Center of the Johannes-Gutenberg University Mainz, Mainz, Germany

**Keywords:** renal fibrosis, ischemia–reperfusion, epithelial–mesenchymal transition, macrolactone, chronic kidney disease

## Abstract

**Background:** Renal fibrosis is one of the most important triggers of chronic kidney disease (CKD), and only a very limited number of therapeutic options are available to stop fibrosis progression. As fibrosis is characterized by inflammation, myofibroblast activation, and extracellular matrix (ECM) deposition, a drug that can address all these processes might be an interesting therapeutic option.

**Methods:** We tested *in vivo* in an ischemia–reperfusion (I/R) model in C57BL/6 mice and in kidney tubular epithelial cells (TEC) (HK2 cell line and primary cells) whether the natural product oxacyclododecindione (Oxa) reduces fibrosis progression in kidney disease. This was evaluated by Western blot, mRNA expression, and mass spectrometry secretome analyses, as well as by immunohistochemistry.

**Results:** Indeed, Oxa blocked the expression of epithelial–mesenchymal transition marker proteins and reduced renal damage, immune cell infiltration, and collagen expression and deposition, both *in vivo* and *in vitro*. Remarkably, the beneficial effects of Oxa were also detected when the natural product was administered at a time point of established fibrotic changes, a situation close to the clinical situation. Initial *in vitro* experiments demonstrated that a synthetic Oxa derivative possesses similar features.

**Conclusion:** Although open questions such as possible side effects need to be investigated, our results indicate that the combination of anti-inflammatory and anti-fibrotic effects of Oxa make the substance a promising candidate for a new therapeutic approach in fibrosis treatment, and thus in the prevention of kidney disease progression.

## 1 Introduction

Acute kidney injury (AKI) is a widespread common clinical complication directly associated with patient morbidity and mortality (Singbartl and Kellum, 2012; Yang, 2019). AKI predisposes to the development and progression of chronic kidney disease (CKD). The concept of AKI–CKD transition has been established in previous clinical and experimental studies (Fiorentino et al., 2018). The incidence rate of AKI has increased in the last decades, resulting in CKD as a global burden and health problem (Lameire et al., 2013). Approximately 850 million people currently suffer from CKD, many of which progress into end-stage kidney disease (ESKD) requiring dialysis or kidney transplantation (Hill et al., 2016; Valdivielso et al., 2019; Gandjour et al., 2020; Sundstrom et al., 2022).

Renal fibrosis as a result of AKI is the common denominator of CKD. It affects all kidney compartments: the tubulointerstitium, glomeruli, tubular epithelium, and vessels as arteriosclerosis (Lopez-Novoa et al., 2011). It is the result of a disturbed homeostasis between injury and repair processes of the renal tissue. The fibrotic changes are characterized by an accumulation of extracellular matrix (ECM) composed of different types of collagen (Lameire et al., 2013; Fiorentino et al., 2018; Yang, 2019), proteoglycans, and glycoproteins (Zhao et al., 2022). A frequent cause of AKI is ischemia–reperfusion (I/R), so this model is well-suited to investigate possible therapeutic effects and the underlying mechanisms of renal fibrosis *versus* repair. In ischemia–reperfusion, increased production of reactive oxygen species and inflammatory processes are considered to be the main trigger for fibrotic changes in the kidney tissue (Panizo et al., 2021; Lopez-Novoa et al., 2011; Hill et al., 2016; Valdivielso et al., 2019; Gandjour et al., 2020; Panizo et al., 2021; Sundstrom et al., 2022; Zhao et al., 2022).

During ischemia–reperfusion (I/R), reperfusion itself causes damage by local inflammation and oxidative stress, contributing to reversible and irreversible changes in tissue viability and organ function (Black et al., 2019). As part of the inflammatory reaction, various immune cells, such as macrophages or T cells, infiltrate the kidney, which mediate the maintenance of inflammation on the one hand and the initiation of fibrotic processes on the other hand. In consequence, various cell types of the kidney are activated, such as mesangial cells, fibroblasts, pericytes, endothelial cells, or tubular epithelial cells (TEC), which themselves produce a complex mixture of pro-inflammatory (TNF-α, IL-1, etc.) and profibrotic mediators (TGF-β, PDG, FGF-2, etc.) and thus actively contribute to the disease process (Black et al., 2019). A typical feature of fibrotic changes in the kidney is the TGF-β-induced formation of myofibroblasts that are major producers of ECM. Myofibroblasts probably arise from TEC (epithelial–mesenchymal transition, EMT) and/or interstitial fibroblasts (Sisto et al., 2021).

However, renal fibrosis itself is not intrinsically progressive after one hit; progression requires additional factors such as repeated or severe episodes of AKI, hemodynamically or inflammatory mediated processes that damage glomeruli and the interstitium (Venkatachalam et al., 2015; Dong et al., 2019). Here, the divergent etiology of AKI to CKD models affects the kidney microenvironment and outcome (fibrosis versus repair). This should be considered in studies evaluating therapeutic options to inhibit the progression of AKI to CKD (Black et al., 2018).

Therapeutic options for CDK are limited and mostly restricted to symptomatic treatment, such as hypertension or blood glucose control, to slow down disease progression. A detailed understanding of the pathogenesis of the acute and chronic kidney damage mechanisms is a prerequisite for finding new therapeutic strategies.

Natural products, discovered in fungi, plants, or microorganisms, such as ciclosporin or tacrolimus, are successfully used in the clinic. Therefore, screening of natural products can be a valuable source to identify new therapeutics (Newman and Cragg, 2020). Oxacyclododecindione (Oxa) is a macrocyclic lactone isolated from the imperfect fungus *Exserohilum rostratum* as a potent inhibitor of IL-4 signaling (Erkel et al., 2008). In addition, a potent anti-inflammatory and TGF-β inhibitory effect with IC_50_ values in the nanomolar range was demonstrated *in vitro* in cell culture experiments (Rudolph et al., 2013; Tauber et al., 2015). Moreover, in MRL/Fas^lpr^ mice, a murine SLE model, Oxa treatment significantly ameliorated glomerulonephritis and reduced collagen deposition in the kidney, indicating an anti-inflammatory and-fibrotic effect of Oxa (Henke et al., 2014).

Therefore, Oxa might be an interesting drug candidate for reducing fibrotic kidney damage. We performed *in vivo* models of I/R-induced kidney injury and *in vitro* cell culture experiments to prove this hypothesis.

## 2 Methods

See Supplementary Material for detailed information.

### 2.1 Animal experiments

We purchased female C57BL/6 (B6) mice (6 weeks of age, 18 ± 1 g). All mice were housed in accordance with standard animal care requirements. The animal studies were approved by the ethical board (23 177-07/G 16-1-021) and performed in accordance with the German animal protection law and the guidelines for the use of experimental animals as stipulated by the Guide of Care and Use of Laboratory Animals of the National Institutes of Health.

### 2.2 Ischemia–reperfusion (I/R)

Mice were anesthetized with 1.5%–2.5% isoflurane added to the respiratory air. We induced unilateral ischemia of the right kidney by clamping the renal pedicle with nontraumatic microaneurysm clamps (Roboz Surgical Instrument Co.) for 45 min. Oxa (1 mg/kg) or dexamethasone (2.5 mg/kg) was applied every other day by intraperitoneal injection for 7 days, starting immediately after I/R-induced kidney injury or after 1 week of I/R injury. PBS/10% EtOH was used as a vehicle. Seven or 20 days after I/R injury, mice were euthanized.

### 2.3 Compounds

Oxa was isolated from fermentations of the imperfect fungus *E. rostratum*, as previously described (Erkel et al., 2008). The purity of Oxa, as estimated by HPLC-DAD/MS analysis, was greater than 99%. 14-Deoxy-14-methyloxacyclododecindione was prepared as described recently (Weber et al., 2020).

### 2.4 Renal histopathology

Kidney pathology was assessed as described previously (Menke et al., 2011). Briefly, kidneys were fixed in 10% neutral buffered formalin for 24 h and embedded in paraffin. Stained paraffin sections (4 μm) with periodic acid–Schiff reagent (PAS) or hematoxylin and eosin (HE) were assessed. Finally, the evaluated scores were summed and divided by the number of high-power fields (HPF) counted and reported as the total score.

### 2.5 Sirius red/Goldner staining

The Institute of Pathology of the Medical Center of the Johannes Gutenberg University performed the Goldner, PAS, and HE staining according to the routinely used protocols. Sirius Red staining was performed with 4-µm paraffin sections and picro-Sirius Red in a saturated aqueous solution of picric acid (Sigma, Deisenhofen, Germany). Evaluation of both stains was performed quantitatively using ImageJ (https://imagej.nih.gov/ij/index.html; version 1.53c).

### 2.6 Immunostainings

Kidney tissue was processed and stained for the presence of CD4, CD8a, CD68, and F4/80 (Supplementary Table S1), as described previously for the kidney (Henke et al., 2014).

### 2.7 Analysis of mRNA expression in kidneys of C57BL/6 (B6) mice and human tubular epithelial cells

To analyze the mRNA expression of different immune relevant genes in cells or mouse tissue, we prepared total RNA by homogenizing the sample in guanidinium isothiocyanate buffer. Gene expression was quantified in a two-step real-time RT-PCR (qRT-PCR) as previously described (Schmidtke et al., 2021) with the oligonucleotides listed in Supplementary Table S2. Specific mRNA expression was normalized to RNA polymerase II (Pol2A) or TATA box-binding protein (TBP) mRNA expression. The 2[ΔΔC(T)] method was used to calculate the relative mRNA expression (Livak and Schmittgen, 2001).

RNA from HK2 cells was isolated using TRIzol (Thermo Fisher Scientific, Waltham, United States) according to the manufacturer’s instructions. Gene expression was quantified using the 5x HOT FIREPoly^®^ EvaGreen^®^ qPCR Supermix (Solis Biodyne, Tartu, Estonia) with the primers listed in Supplementary Table S2. Relative mRNA amounts were determined using the mathematical model for relative quantification in real-time PCR proposed by Pfaffl (2001).

### 2.8 Tissue preparation and cell culture

Human TECs were isolated from a piece of the kidney (discarded healthy tissue from nephrectomies) in the same manner as described previously (Faust et al., 2002). Stimulation was performed with a cytokine mix (CM) [IFN-γ (300 U/ml), IL-1β (600 U/ml), and TNF-α (37 ng/ml)] for 2 or 24 h, ethics approval numbers 837.467.13 and 2019-14695.

### 2.9 Transient transfection and cell viability

The Smad binding reporter plasmid (AGCCAGACA) 9MLP-Luc (Dennler et al., 1998) was co-transfected with the control reporter vector pRL-EF1α in HK2 cells. For induction of luciferase expression, cells were treated with 5 ng/mL TGF-β for 24 h. The luciferase activity was measured using the Dual-Glo Luciferase assay system (Promega, Mannheim, Germany) according to the manufacturer’s instructions. A Giemsa stain-based cell viability assay was performed after 48 h, as previously described (Mirabelli et al., 1985), to evaluate the effect of Oxa on cellular proliferation and viability.

### 2.10 HK2 cell stimulation

As a cellular model, the human tubule epithelial cell line human kidney 2 (HK2, ATCC CRL-2190) was used. 70%–80% confluent HK2 cells were starved in a medium containing 0.5% FCS for 24 h, pretreated for 1 h with or without different concentrations of test compound, and induced with 5 ng/ml TGF-β as indicated (24 h: reporter gene assay, 48 h: Western blot; 7 h: qRT-PCR). Total cell extracts were prepared for RNA or protein isolation.

### 2.11 Western blot

Proteins were separated using 10% SDS-PAGE and detected with N-cadherin (D4R1H, 13116), E-cadherin (24E10, 3195), Snail (C15D3, 3879), α-tubulin (11H10; 2125), and acetyl-α-tubulin (D20G3, Lys40; 5335) (all Cell Signaling Technology, Danvers, United States) antibodies. Antibodies against β-actin (13E5; 4970, Cell Signaling Technology, Danvers, United States) served as an endogenous control.

### 2.12 Zymography

Serum-starved HK2 cells were stimulated as described above. The cell culture supernatant was analyzed for the activity of matrix metalloproteinases using gelatin-zymography as described previously (Kupai et al., 2010). ImageJ software (http://rsb.info.nih.gov/ij) was used for quantification.

### 2.13 Mass spectrometry

HK2 cells were stimulated, and the supernatant was prepared as described for zymography. Mass spectrometry analysis was performed on a high-resolution LC-MS system (Eksigent nanoLC 425 coupled to a Triple-TOF 6600, AB Sciex) in an information-dependent acquisition (IDA) mode. For detailed information, see Supplementary Material.

### 2.14 Statistics

Data represent means ± SEM. Statistical differences were determined using factorial analysis of variance followed by “Tukey’s” or “Dunnett’s” multiple comparison tests. In the case of two means, classical *t*-test analyses were used. Two-way ANOVA analysis was performed, followed by Bonferroni’s multiple comparisons test. All statistical analyses were performed using GraphPad Prism 9.0.

## 3 Results

### 3.1 Oxa treatment ameliorates kidney damage following I/R

**FIGURE 1:**
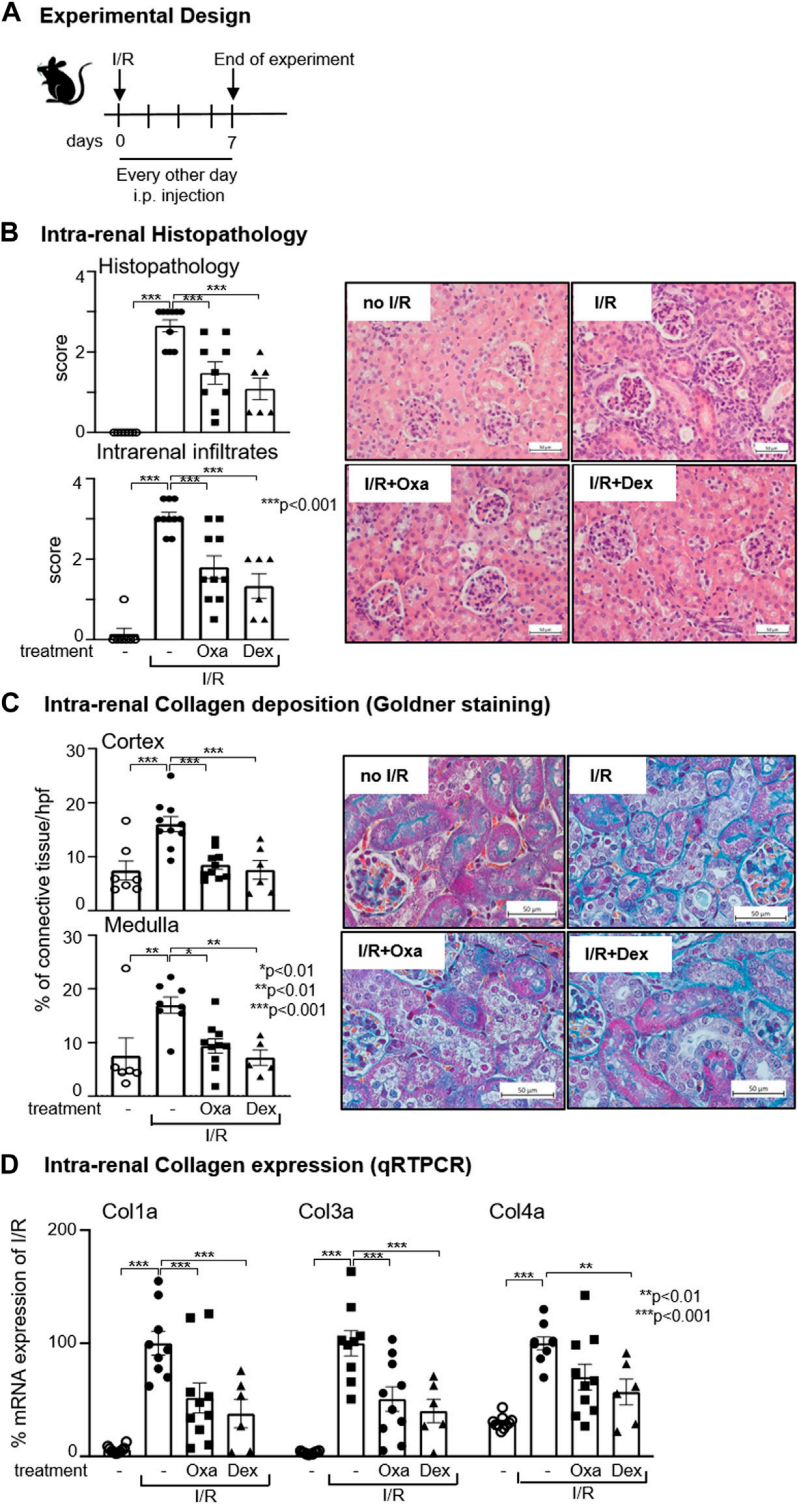
Oxa treatment ameliorates kidney damage following I/R. **(A)** Experimental setup. Six-week-old C57BL/6 (B6) mice were subjected to I/R injury and subsequently treated with 1 mg/kg oxacyclododecindione (I/R Oxa) (*n* = 10), 2.5 mg/kg dexamethasone (I/R Dex) (*n* = 6), or vehicle (I/R) (*n* = 10) every other day by intraperitoneal injection. Seven days after I/R injury, mice were euthanized. Untreated (-) mice (*n* = 7) served as control. **(B)** I/R-induced renal injury and cell infiltration were assessed by hematoxylin–eosin (HE) and periodic acid–Schiff (PAS) staining and were scored blinded in a semi-quantitative manner. Representative microphotographs of HE staining are shown. **(C)** Representative microphotographs demonstrate collagen deposition by Masson Goldner staining, which was scored quantitatively with ImageJ (Version 1.53c) software. **(D)** Data show the relative renal collagen 1a, 3a, and 4a mRNA expression normalized to the expression of the housekeeping gene RNA polymerase IIa evaluated using quantitative real-time RT-PCR (qRT-PCR). All data are expressed as mean (±SEM) compared to I/R-treated mice (***p < 0.001; **p < 0.01; vs. I/R-treated mice) by one-way analysis of variance with *post hoc* Dunnett’s multiple comparison test.

Previous *in vitro* and *in vivo* data demonstrated an anti-inflammatory effect of Oxa and suggested an anti-fibrotic activity of the natural product (Rudolph et al., 2013; Henke et al., 2014; Tauber et al., 2015). Therefore, we were interested in the effects of Oxa on renal pathology and tested its effects in an I/R model leading to renal injury characterized by inflammation, repair processes, and fibrotic tissue changes, resulting in impaired renal function. In the first experimental setup, mice were treated every other day for 7 days immediately after unilateral I/R of the right kidney with Oxa or dexamethasone (Scheme, Figure 1A). In our model, the glomerular and interstitial injury and the number of infiltrating cells were reduced in I/R kidneys by Oxa treatment (Figure 1B). The effect of Oxa treatment was comparable to that of dexamethasone, indicating a protective effect of the macrocyclic lactone.

A hallmark of fibrosis development is the increased deposition of ECM. Goldner staining [or Sirius Red (data not shown)] demonstrated less deposition of ECM in the kidney (medulla and cortex) of Oxa-treated C57BL/6 mice upon I/R injury compared to control mice (Figure 1C). Quantification revealed that Oxa treatment reduced collagen deposition to roughly half of the I/R control. The I/R-mediated increase in the mRNA expression level of collagen types 1a, 3a, and 4a, all known to be involved in fibrosis-mediated ECM deposition, was reduced by Oxa treatment. The effect on collagen type 1a and type 3a mRNA expression was significant (Figure 1D). All Oxa effects were comparable to those of dexamethasone. In summary, our data present evidence that Oxa is able to reduce I/R-induced ECM deposition.

### 3.2 Oxa treatment results in reduced numbers of intrarenal macrophages and prevents their shift to profibrotic M2 macrophages

**FIGURE 2:**
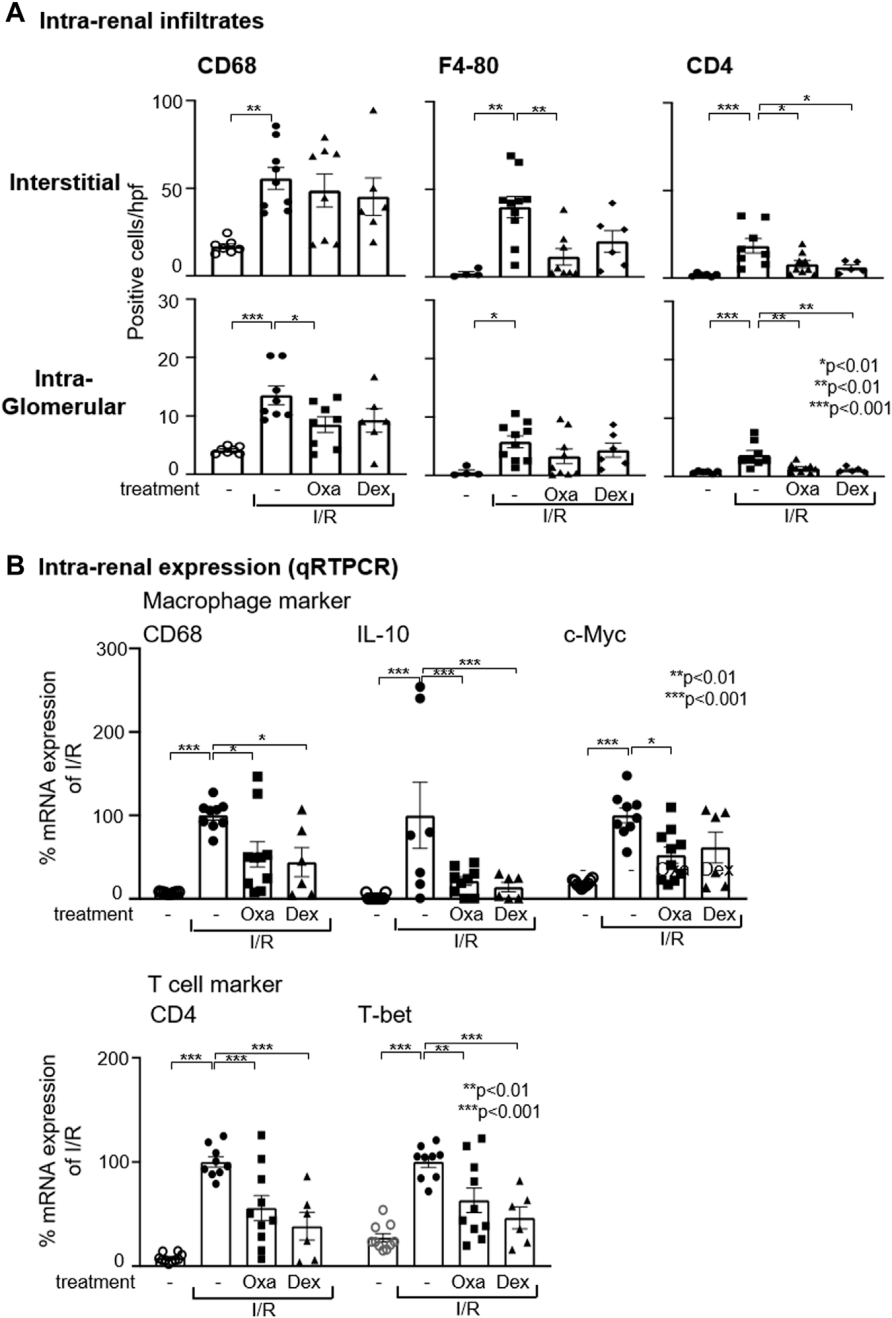
Oxa treatment reduces intra-renal macrophage and T-cell infiltration. **(A)** Interstitial and intra-glomerular macrophage and T-cell abundance was determined by CD68, F4-80, and CD4 T cell immunostaining in untreated mice and vehicle- (I/R) (*n* = 10), Oxa- (I/R Oxa) (*n* = 10), and Dex- (I/R Dex) (*n* = 6) treated mice. **(B)** Intra-renal relative mRNA expression of CD68, IL-10, c-Myc, CD4, and T-bet was evaluated by qRT-PCR. The mRNA expression data were normalized to the expression of the housekeeping gene RNA polymerase IIa. All data are expressed as mean (±SEM) compared to I/R-treated mice (***p < 0.001; **p < 0.01; *p < 0.05 vs. I/R-treated mice) by one-way analysis of variance with *post hoc* Dunnett’s multiple comparison test.

Given the protective effect of Oxa treatment in I/R damage, mechanisms influenced by Oxa are of interest. CD68 and F4-80 are established markers for monocytes/macrophages that are essentially involved in kidney fibrosis. Oxa treatment ameliorated I/R-induced intra-renal infiltration of CD68- and F4-80-positive cells (Figure 2A) and CD68 mRNA expression (Figure 2B).

In the pathogenesis of fibrosis, the disturbed balance of M1 and M2 macrophages plays a causative role. Simplified, M1 macrophages have pro-inflammatory functions, whereas M2 macrophages are important for repair and wound healing processes. According to qRT-PCR analyses, I/R led to an increase in the mRNA expression of IL-10 and c-myc, two M2 markers, while Oxa treatment caused a strong reduction in IL-10 mRNA expression and reduced c-myc mRNA by about 50%. This may indicate that Oxa is able to inhibit M2 macrophages (Figure 2B).

In addition to macrophages, T cells are also drivers of fibrosis development. Immunohistochemistry staining and qRT-PCR results demonstrated that Oxa treatment attenuated CD4^+^ T cell infiltration induced by I/R (Figures 2A, B). Dexamethasone effects were comparable to those of Oxa. Further characterization of infiltrating T cell subpopulation revealed enhanced T-bet mRNA expression, a transcription factor known as T helper (Th) cell 1 marker in the kidney of I/R-treated mice (Figure 2B). This increase was partially reversed by Oxa and dexamethasone treatment. These data indicate that primarily Th1 cells infiltrate into the kidney upon I/R injury and drive fibrotic changes.

### 3.3 Oxa treatment inhibits profibrotic changes by inhibition of EMT-associated changes

**FIGURE 3:**
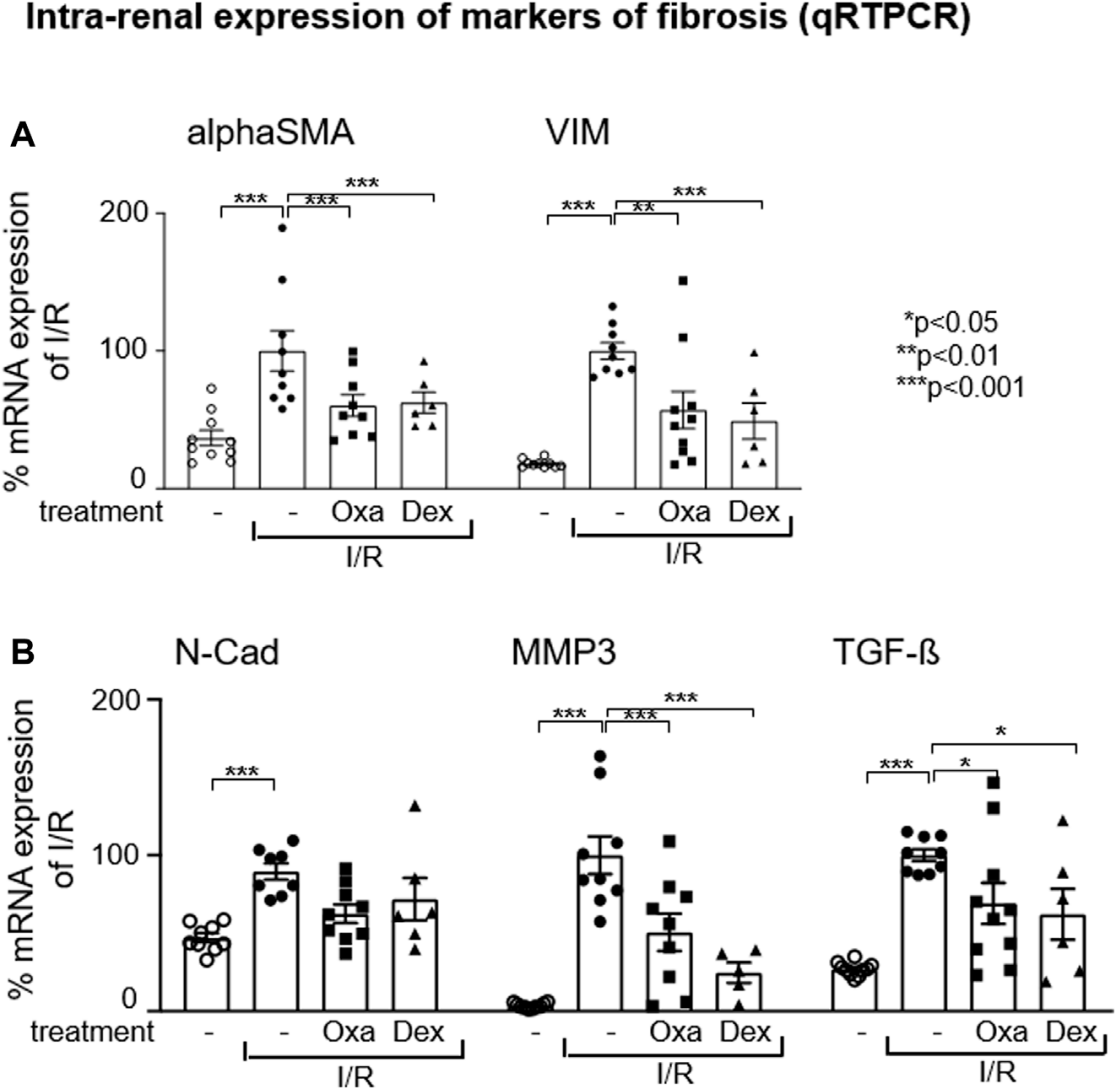
Oxa treatment reduces the expression of EMT and fibrosis-associated marker genes. **(A,B)** Intra-renal relative mRNA expression of alpha-smooth muscle actin (alpha SMA), vimentin (Vim), N-cadherin (N-Cad), matrix metalloproteinase 3 (MMP3), and TGF-beta (TGF-β) was determined in untreated mice (*n* = 10) and vehicle- (I/R) (*n* = 9), Oxa- (I/R Oxa) (*n* = 10) and Dex- (I/R Dex) (*n* = 6) treated mice by qRT-PCR. Data were normalized to the expression of the housekeeping gene RNA Polymerase IIa. All data are expressed as mean (±SEM) compared to I/R-treated mice (***p < 0.001; **p < 0.01; *p < 0.05 vs. I/R-treated mice) by one-way analysis of variance with *post hoc* Dunnett’s multiple comparison test.

It has been described that during epithelial–mesenchymal transition, injured renal tubule epithelial cells change their phenotype to α-smooth muscle actin (α-SMA)^+^ myofibroblasts, which appear to be the major type of matrix-producing cells that drive fibrogenesis in renal disease (Sheng and Zhuang, 2020). Therefore, we checked in our I/R model the expression of EMT marker genes. I/R injury-induced mRNA expression of filament protein vimentin (Vim) and αSMA, both key markers of EMT necessary to initiate cell migration, reorganization, and contractile function, indicates profibrotic changes in the injured kidney (Figure 3A). This effect was reversed by Oxa and dexamethasone treatment and supports the hypothesis that the natural product is able to inhibit EMT-associated changes effectively. The mRNA expression of the EMT marker N-cadherin (N-Cadh) was induced in the I/R-treated mouse group, but this effect was not significantly altered by Oxa or dexamethasone. In contrast, I/R-induced matrix metalloproteinase (MMP) 3 and TGF-β mRNA expression were reduced by Oxa in a comparable fashion to that of dexamethasone (Figure 3B).

### 3.4 In a therapeutic approach, Oxa reduces already-established fibrosis in the kidney

**FIGURE 4:**
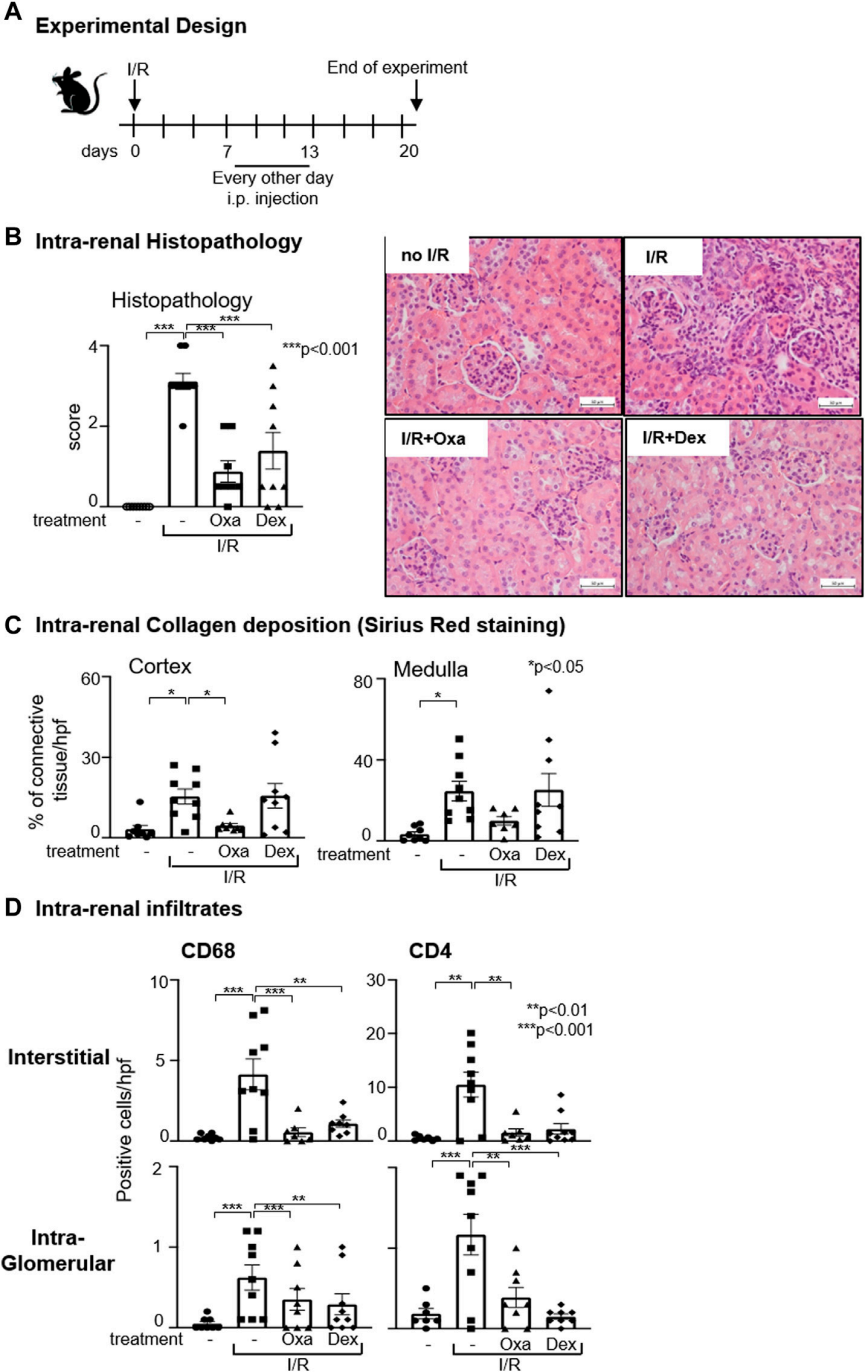
Oxa treatment ameliorates kidney damage after the onset of I/R. **(A)** Experimental setup. Six-week-old C57BL/6 mice were subjected to I/R injury (I/R-) (*n* = 9). On day 7, 1 mg/kg Oxa (I/R Oxa) (*n* = 8) or 2.5 mg/kg dexamethasone (I/R Dex) (*n* = 9) was administered every other day by intraperitoneal injection for 7 days until day 13. Untreated mice served as negative control (−) (*n* = 8). All mice were euthanized on day 20, 7 days after the last drug application. **(B)** I/R-induced renal injury was assessed by HE and PAS staining. Renal damage of kidneys without (−) or with I/R (I/R) was compared with kidneys subjected to I/R and Oxa (I/R Oxa) or I/R and Dex (I/R Dex) treatment. Representative microphotographs of HE staining. **(C)** Intra-renal collagen deposition was determined with Sirius Red staining and quantified with ImageJ (Version 1.53c) software. **(D)** Interstitial and intra-glomerular macrophage and T-cell abundance was determined by CD68 and CD4 immunstaining. All data are expressed as mean (±SEM) compared to I/R-treated mice (***p < 0.001; **p < 0.01; vs. I/R-treated mice) by one-way analysis of variance with *post hoc* Dunnett’s multiple comparison test.

To further characterize the anti-fibrotic properties of macrocyclic lactones, we tested the potency of Oxa to diminish already established I/R-induced fibrotic changes in the kidney. Therefore, we administered Oxa from day 7 to day 13 after unilateral I/R injury of the right kidney, when fibrotic changes as EMT and enhanced ECM deposition already appeared (Scheme, Figure 4A). In the kidney, we detected at day 20 following I/R injury increased tissue damage accompanied by increased collagen deposition compared to the control group (Figures 4B, C). Both were reversed by Oxa treatment, indicating that the natural product is able to attenuate established fibrotic changes in the cortex and medulla of the kidney. Moreover, I/R-induced infiltration of CD68^+^ monocytes/macrophages and CD4^+^ T cells was nearly reversed to the level of untreated control animals by Oxa treatment. In contrast to collagen deposition, where dexamethasone was less effective, CD68^+^ cell infiltration was also reduced by the glucocorticoid.

### 3.5 Oxa modifies human tubular epithelial cell activity

**FIGURE 5:**
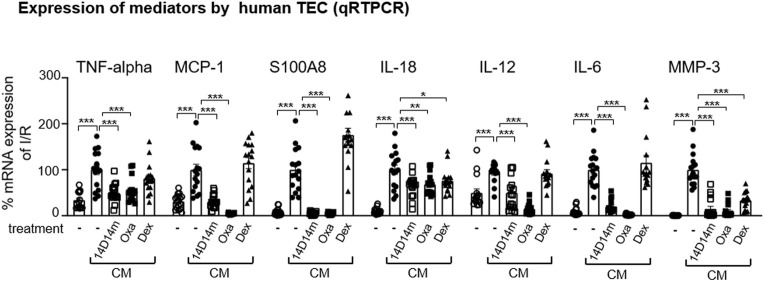
Oxa treatment reduces the expression of kidney damage-associated marker genes in human TEC. Human TECs were stimulated with a cytokine mixture [CM: IFN-γ (300 U/ml), IL1β (600 U/ml), and TNF-α (37.5 ng/ml)] for 2 and 24 h. 1 h before CM stimulation, cells were treated with 100 ng/ml 14-deoxy-14-methyloxa (14D14m), 100 ng/ml Oxa (Oxa) or 50 μg/ml dexamethasone (Dex). The relative mRNA expression of TNF-α was determined after 2 h CM stimulation, and MCP-1, S100A8, IL-18, IL-12, IL-6, and MMP-3 mRNA expression was measured after 24 h CM incubation. Data were normalized to the expression of the housekeeping gene RNA polymerase IIa. All data are expressed as mean (±SEM) compared to CM-treated TEC (***p < 0.001; **p < 0.01; *p < 0.05 vs. CM-treated TEC) by one-way analysis of variance with *post hoc* Dunnett’s multiple comparison test, *n* = 4 TEC of different donors each with four technical replicates.

To dissect Oxa effects on intra-renal mechanisms that mediate fibrosis, we evaluated the possible role of Oxa on human tubular epithelial cells (hTEC), which are activated during kidney injury and contribute to fibrotic changes by production of pro-inflammatory and profibrotic mediators. Moreover, the availability of synthetic Oxa-derivatives with comparable biological activities to Oxa is a prerequisite for further research. Therefore, we tested in the hTEC cell culture model besides Oxa, also the effect of 14-deoxy-14-methyloxacyclododecindione, a synthetic Oxa-derivative. qRT-PCR experiments demonstrated that cytokine-induced expression of TNF-α, MCP-1, S100A8, IL-18, IL-12, and IL-6 in hTEC was inhibited by Oxa (100 ng/mL) and 14-deoxy-14-methyloxa (100 ng/ml) (Figure 5). A similar effect was seen for MMP3. It is striking that in contrast to both macrocyclic lactones, dexamethasone (50 μg/ml) did not reverse the mRNA expression of most of these inflammatory genes. In conclusion, macrocyclic lactones seem to be able to modify the hTEC activity.

### 3.6 Oxa effects on TGF-β signaling, cell viability, and EMT-associated processes

**FIGURE 6:**
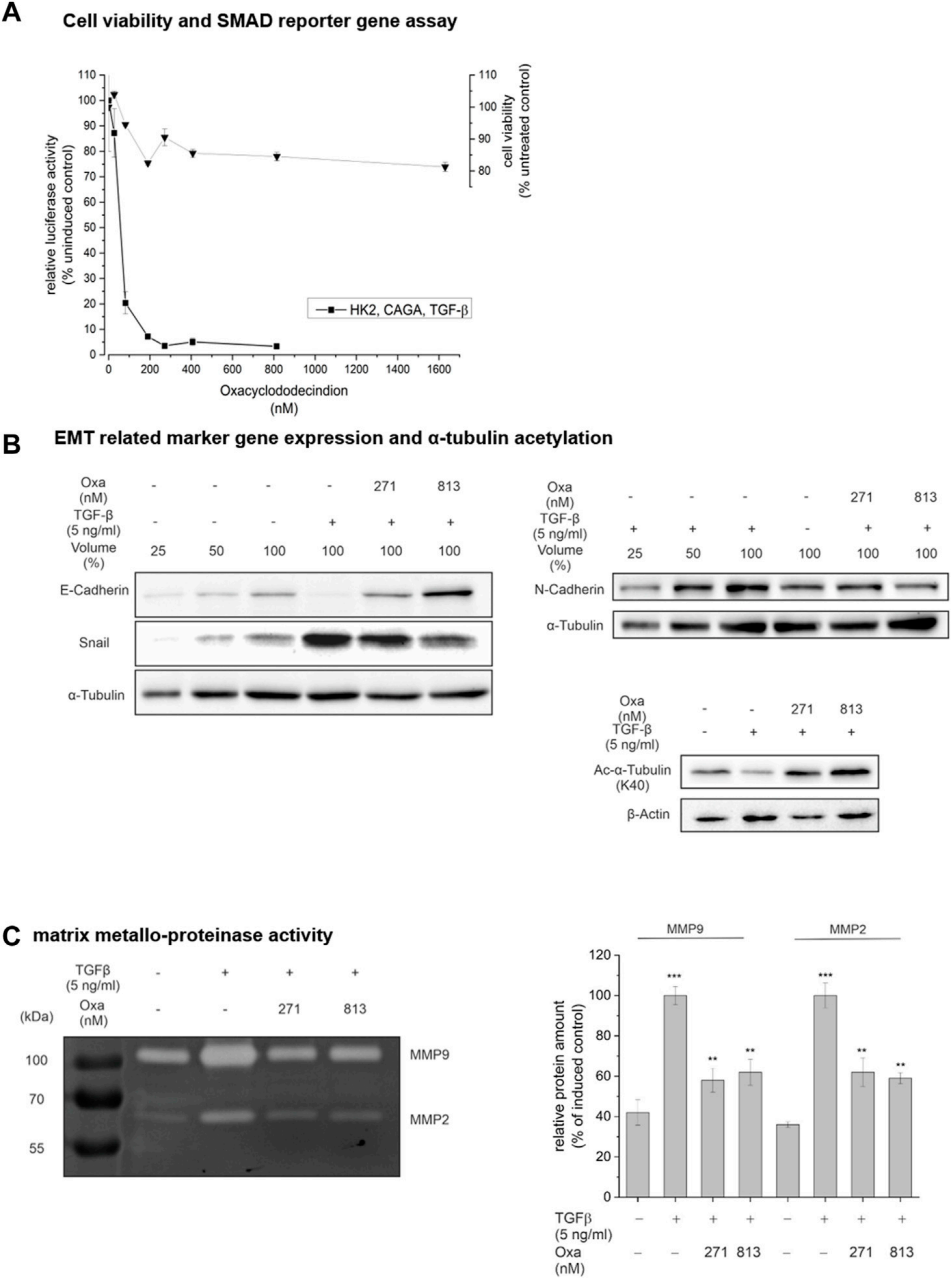
Effect of Oxa on TGF-β signaling, cell viability, and EMT-associated processes. **(A)** HK2 cells were transiently transfected with a transcriptional reporter driving luciferase expression from the adenovirus major late promoter under the control of nine tandem copies of the CAGA Smad2/3 binding element [(AGCCAGACA)_9_MLP-Luc] together with a constitutive EF1α-promoter-dependent reporter as internal control, pretreated for 1 h with Oxa and stimulated with 5 ng/ml TGF-β for 24 h. Control (100%): stimulation only. Data represent the mean ± SEM of at least three independent experiments. Cytotoxicity was measured by Giemsa staining. **(B)** Effect of Oxa on the expression of EMT-related marker genes and a-tubulin acetylation and **(C)** on the activity of secreted matrix metalloproteinases in HK2 cells. HK2 cells were pretreated with the indicated concentrations of Oxa for 1 h prior to stimulation with 5 ng/ml TGF-β for 48 h. Protein content in whole cell lysates (25–100 µg total protein extract) was analyzed using Western blotting. Results are a representative experiment repeated three times with essentially similar findings. **(C)** Detection of MMP2 and MMP9 activity of 25 µL 10-fold concentrated cell culture supernatant by gelatin zymography. Representative gel of three individual experiments. Densiometric analysis was performed with ImageJ software and presented relative protein amount ± SEM (***p < 0.001; **p < 0.01 vs. unstimulated cells).

To gain mechanistic insight into the mode of Oxa action on renal pathology, we used the human tubular epithelial cell line human kidney 2 (HK2) as a cell culture model. The primary goal of these experiments was to determine whether Oxa modulates critical mediators of fibrogenesis, such as TGF-β mediated signaling pathways or the EMT process. First, the effects of Oxa on cell viability and growth were determined. The Giemsa stain-based cell viability assay demonstrated a minor, 15% reduced cell growth of HK2 cells upon Oxa treatment, indicating slight growth retardation but no major cytotoxic Oxa effects (Figure 6A). Subsequently, HK 2 cells were transiently transfected with a luciferase reporter gene where luciferase expression depends on the binding of TGF-β-mediated, activated SMAD2/3 transcription factors. Even at low nanomolar concentrations (100–271 nM), Oxa almost completely blocked TGF-β-SMAD-dependent luciferase expression. Therefore, Oxa seems to be a potent inhibitor of TGF-β induced signaling pathways (Figure 6A). As TGF-β is an important profibrotic factor known to induce EMT, a hallmark of fibrotic changes, we tested in HK2 cells whether Oxa is able to modify the expression of EMT-associated marker proteins. Indeed, 271 and 813 nM Oxa restored TGF-β-mediated downregulation of the epithelial marker protein E-cadherin (E-cadh) (Figure 6B). In accordance, Oxa suppressed TGF-β-induced expression of the transcription factor Snail, which is involved in EMT induction, and of N-cadherin, a mesenchymal cell marker. Loss of α-tubulin acetylation, another marker of TGF-β-induced EMT (Gu et al., 2016), was reverted upon Oxa treatment (Figure 6B). Moreover, Oxa reduced TGF-β-induced MMP 2 and 9 activity, which are involved in EMT (Figure 6C). Altogether, these experiments present evidence that Oxa is able to inhibit the expression of EMT-associated markers in HK2 cells, even at low nanomolar concentrations.

**FIGURE 7:**
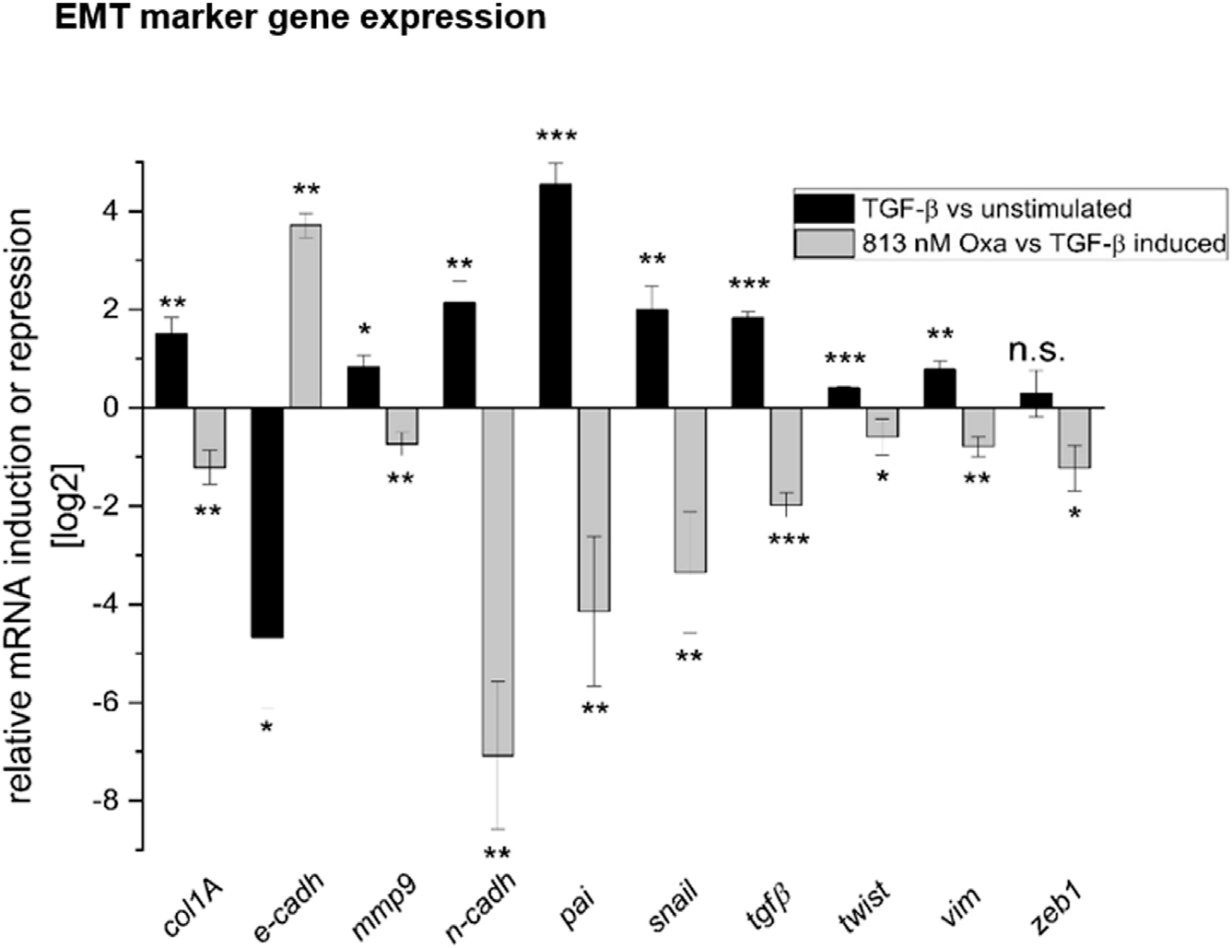
Oxa treatment reduces the expression of EMT and fibrosis-associated marker genes. Effect of Oxa on mRNA levels of selected TGF-β inducible genes in HK2 cells. Values are expressed as ratios (log_2_) of relative mRNA levels of stimulated (7 h TGF-β) versus un-stimulated cells as control and compound pretreated and stimulated versus untreated, stimulated cells, corrected for GAPDH as reference determined in the same sample in parallel. Data are shown as mean values ± SEM of three independent experiments (***p < 0.001; **p < 0.01; *p < 0.05; n.s, not significant vs. stimulated cells). Collagen type 1A (col1A), E-cadherin (E-cadh), matrix metalloproteinase 9 (MMP9), N-cadherin (N-Cad), plasminogen-activator-inhibitor (pai), Snail (snail), TGF-beta (TGF-β), twist, vimentin (Vim), and Zinc finger E-box binding homeobox 1 (zeb1) were determined.

mRNA expression analyses of EMT marker genes in HK2 cells showed that Oxa restored TGF-β-mediated repression of E-cadh mRNA nearly to the control level. Furthermore, mRNA expression of EMT-associated factors, such as collagen type 1A (col1A), MMP9, N-Cadh, plasminogen-activator-inhibitor (pai), Snail (snail), TGF-β, Twist (twist), vimentin, and zinc finger E-box binding homeobox 1 (zeb1), was significantly reduced by Oxa treatment (Figure 7). Altogether, these first experiments present evidence that Oxa is able to inhibit the expression of EMT-associated markers in HK2 cells. Therefore, it may be that these mechanisms mediated by Oxa are responsible for the reduction in fibrotic changes observed in our *in vivo* model.

### 3.7 Influence of OXA on the secretome of HK2 cells

**FIGURE 8:**
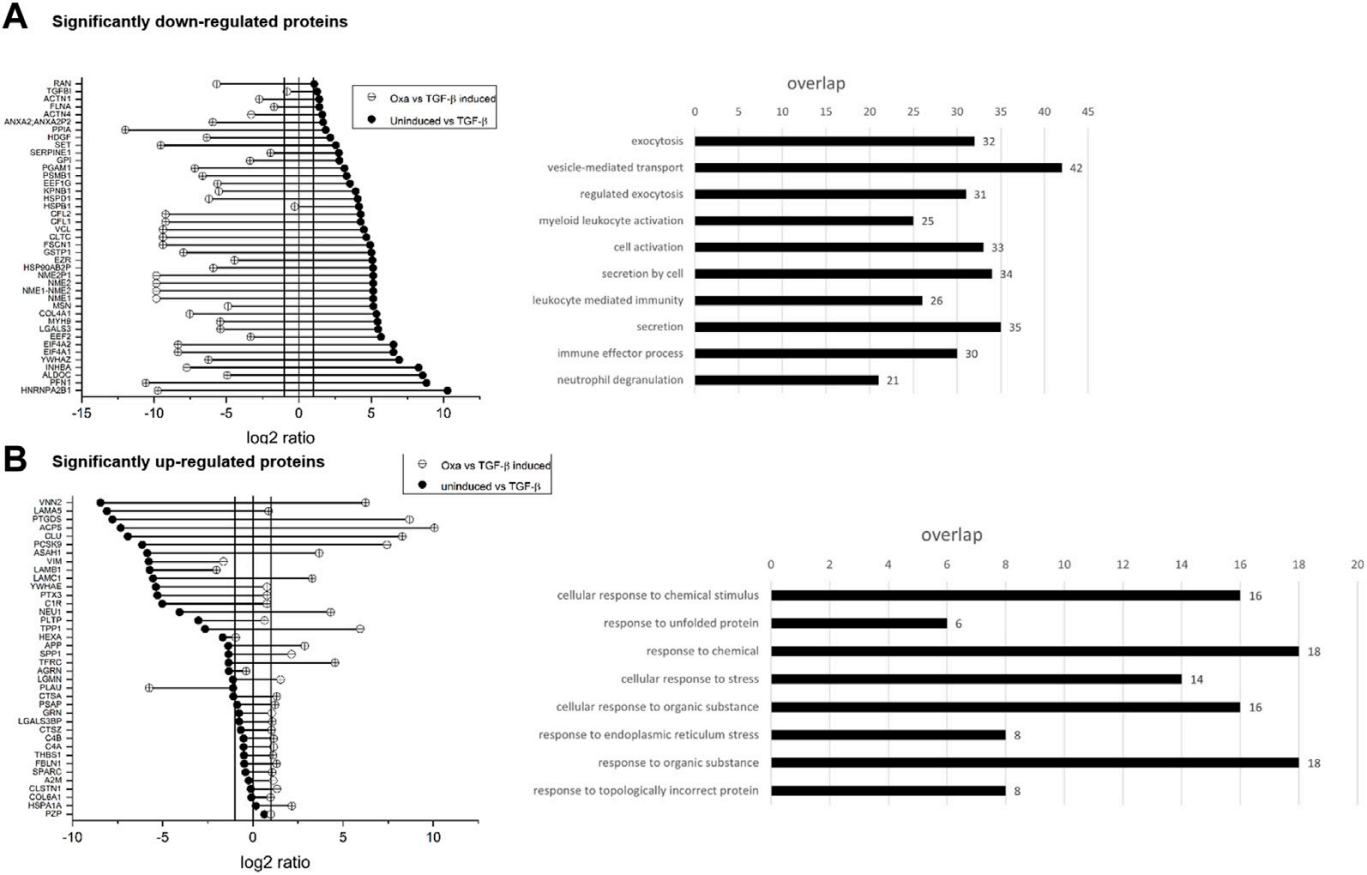
Influence of OXA on the secretome of HK2 cells. **(A,B)** Values are expressed as ratios (log_2_) of relative secreted protein levels of stimulated (48 h, 5 ng/ml TGF-β) versus un-stimulated cells as control and TGF-β-stimulated cells with or without Oxa pre-treatment (813 nM). Data are shown as mean values of four independent experiments. Significantly down- and **(A)** upregulated **(B)** proteins in TGF-β stimulated HK2 cells after Oxa treatment and overrepresented GO terms (*p* < 0.01). Bar length represents the number of proteins in different functional groups.

As we demonstrated previously, Oxa inhibits the transcription of ECM and EMT components and ameliorates I/R-induced kidney fibrosis *in vivo*. To gain insight into the underlying molecular mechanisms of Oxa effects, we analyzed the secretome of TGF-β stimulated (5 ng/ml for 48 h) HK2 cells in the absence or presence of Oxa (813 nM) with protein mass spectrometry. A total of 108 proteins with a *p*-value ≤ 0.05 were identified to be differentially expressed (≥two fold upregulated or 50% downregulated) in the secretome of TGF-β-stimulated cells. The expression of 80 proteins was significantly altered in cells pretreated with Oxa and stimulated with TGF-β for 48 h. Proteins with significant changes in expression were analyzed by Gene Ontology annotations and functional enrichment (molecular function and cellular compartment) using the g:Profiler software (Raudvere et al., 2019). Significantly, Oxa-downregulated proteins were categorized into groups related to secretion, including ECM, exocytosis, vesicle-mediated transport, and different immune effector processes (*p* < 0.01) (Figure 8).

## 4 Discussion

Treatment of fibrosis is still a medical challenge, as only a very limited number of therapeutic options are available, which are not able to entirely stop fibrosis progression or even reverse the pathological changes. However, the development of targeted therapy is of great importance from both a medical and health-economic perspective. Fibrosis is characterized by an imbalance of inflammation and repair processes, driven by TGF-β, EMT, and enhanced ECM deposition (Rastaldi et al., 2002; Kalluri and Weinberg, 2009). Therefore, a lot of potential drug target molecules may exist. Theoretically, fibrosis progression could be prevented by anti-inflammatory mechanisms, the modulation of repair processes, or, even better, both.

We provide evidence that Oxa is able to prevent and ameliorate I/R-induced renal damage, cell infiltration, inflammation, and ECM deposition *in vivo.* Especially, the ability to reduce already established I/R-induced renal damage, which is particularly relevant for a large number of CKD patients, makes Oxa an interesting drug candidate for the treatment of fibrotic changes in acute and chronic kidney disease. Moreover, also in human primary TEC, Oxa seems to have anti-inflammatory and anti-fibrotic effects. *In vitro* data derived from human HK2 cells imply that the Oxa effects seen *in vivo* might rely on Oxa-mediated inhibition of TGF-β activated expression of fibrosis-associated markers involved in EMT and EMC. Oxa inhibited the EMT characteristic N-cadherin switch and reduced the expression of Snail1, a transcription factor responsible for the repression of epithelial marker genes and the induction of EMT-associated mesenchymal markers, therefore contributing significantly to renal fibrosis (Simon-Tillaux and Hertig, 2017). Oxa also reduced TGF-β-induced production and secretion of MMP-2 and MMP-9, which are involved in ECM remodeling during EMT and kidney fibrosis (Cheng et al., 2017). Some effects of Oxa were comparable to those of dexamethasone. Nevertheless, it is very unlikely that Oxa signals via the glucocorticoid receptor pathway because the effects of natural macrolactones are also detectable in cell lines that do not express a glucocorticoid receptor (Schmidt et al., 2010).

Most likely, the effect of reduced fibrosis development is not solely due to the inhibition of EMT by Oxa but is accompanied simultaneously by an effective blockade of the inflammatory signaling cascade (O'Sullivan et al., 2022). In addition to the reduction of leukocytes infiltrating the kidneys, our qRT-PCR results indicate that Oxa might regulate the activity of the so-called alternative M2 macrophages (Wang et al., 2021). This subtype of macrophages actually contributes to the resolution of inflammation. However, these macrophages produce a number of factors, such as TGF-β, PDGF, or FGF-2, which induce the formation of myofibroblasts and thus promote the development of renal fibrosis (Wen et al., 2021). The well-documented anti-inflammatory effect of Oxa might be responsible for the fact that, in parallel also, the activity of M1 macrophages may be attenuated. M1 macrophages secrete pro-inflammatory mediators, induce the infiltration of immune cells at the site of inflammation, and thus contribute to maintaining intra-renal inflammation. Moreover, they stimulate TEZ to form profibrotic mediators (Tang et al., 2019). Oxa-mediated inhibition of both mechanisms (anti-inflammatory and anti-fibrotic) may be the reason for the beneficial effects in I/R-induced renal fibrosis.

Our secretome data identify additional Oxa target proteins that may contribute to the nephroprotective effects of the natural product. Here, Oxa reduced protein expression of collagen type 4 alpha 1, galectin, and serpin1, which modulate ECM deposition, as well as moesin, inhibin beta A subunit, and myosin heavy chain 9, which are known to be involved in SLE and LN pathogenesis (Villanueva et al., 2011; Lin et al., 2012; Chen et al., 2014). Gene Ontology analysis of Oxa-upregulated proteins (>two fold) is categorized into several terms related to cellular stress against chemicals and unfolded protein response. Interestingly, Oxa treatment significantly upregulated the expression of the secreted small heat shock protein clusterin (CLU), which has been shown to protect cells from apoptosis induced by cellular stress (Jones and Jomary, 2002). Studies in mouse models showed that an increased concentration of CLU in serum could protect animals from developing renal fibrosis (Jung et al., 2012). Further experiments are needed to determine whether these are direct Oxa effects or the result of Oxa-mediated modulation of various signaling pathways that remain to be identified.

As the natural product Oxa can only be produced in small quantities by fermentation, synthetic production is decisive for the possible therapeutic use in humans. Here, we show first *in vitro* results of a synthetic derivative of Oxa, the 14-deoxy-14-methyloxa that seems to have similar effects in human TEC as the original natural macrolactone. This is an important step in developing a new lead compound for the development of anti-fibrotic drugs.

Intriguingly, we could demonstrate the combination of anti-inflammatory and anti-fibrotic effects of Oxa in acute and chronic kidney injury. In particular, the results of progression inhibition of fibrosis after established damage are convincing and represent progress in contrast to the currently available drugs. In particular, knowing that fibrosis is often triggered not only by mechanical effects but also by inflammatory processes, Oxa represents, for example, an option for a potentially even more efficient therapy compared to the SGLT-2 inhibitors currently newly approved for the slowing of progression in chronic renal failure (Wheeler et al., 2021; The et al., 2023). The advantage of Oxa is its direct effect on the secretion of anti-fibrotic and anti-inflammatory mediators in renal TEC, whereas the function of SGLT-2 inhibitors is primarily anti-hyperglycemic. The mechanism behind the observed effects of SGLT-2 inhibitors on inflammation and tissue fibrosis can currently not be fully explained and might be indirect via the reduction of reactive oxygen species (Yaribeygi et al., 2019; Xuan et al., 2021). Some murine studies showed similar (Castoldi et al., 2020; Xuan et al., 2021) fibrosis-preventing results after treatment with SGLT-2 inhibitors compared to our results (see Figure 1), whereas others observed a lesser or no anti-fibrotic effect (Gangadharan Komala et al., 2014; Jigheh et al., 2019; Castoldi et al., 2021). The reduction of renal CD68^+^ monocytes/macrophages after treatment with an SGLT-2 inhibitor was less effective (Wang et al., 2018), as shown in this study for Oxa (Figure 4). Moreover, the diminishing effect on IL-6 mRNA expression in an *in vitro* setting (Chi et al., 2022) was not comparable to the inhibition observed for Oxa (Figure 5). Certainly, side effects and long-term effects still need to be evaluated to allow a direct comparison of different compounds to Oxa, especially for translational aspects. Further experiments should prove the efficiency and safety of this drug.

## 5 Conclusion

In summary, our data shed light on the role of Oxa as a potential new therapeutic drug in acute and chronic kidney disease. We demonstrate the attenuation of fibrosis and inflammation in a prophylactic and therapeutic approach in a mouse model and *in vitro* in human cell lines and primary cells. Our work demonstrates the ability of Oxa and its synthetic derivative 14-deoxy-14-methyloxa to prevent EMT and inflammation, leading to a new therapeutic drug option in the future.

## Data Availability

The original contributions presented in the study are included in the article/Supplementary Materials and on https://git.nfdi4plants.org/venn/Antifibrotic-effects-of-Oxa for proteomics data. Further inquiries can be directed to the corresponding authors.

## Abbreviations

ECM, extracellular matrix; EMT, epithelial–mesenchymal transition; ESKD, end-stage kidney disease; I/R, ischemia–reperfusion; MMP, matrix metalloproteinase; Oxa, oxacyclododecindione; TEC, tubular epithelial cells.
