# Supplementary material for: The macrocyclic lactone oxacyclododecindione reduces fibrosis progression
Source: Front Pharmacol. 2023 Jun 13;14:1200164. doi: 10.3389/fphar.2023.1200164 (PMC10294233; doi:10.3389/fphar.2023.1200164)
Supplement: Supplementary file 1 [file DataSheet1.pdf]

Supplemental Material

The macrocyclic lactone Oxacyclododecindione reduces fibrosis progression

Sabrina Saurin*, Myriam Meineck*, Markus Rohr[§], Wilfried Roth[#], Till Opatz[&], Gerhard Erkel[§], Andrea Pautz[Δ$], Julia Weinmann-Menke*[$]

Department of Nephrology, Center of Immunotherapy, [Δ]Institute of Pharmacology, [#]Institute of Pathology, Medical Center of the Johannes-Gutenberg University Mainz, Mainz, Germany.

[&]Department of Chemistry, Johannes-Gutenberg University Mainz, Duesbergweg 10-14, 55128 Mainz, Germany.

[§]Department of Molecular Biotechnology & Systems Biology, RPTU Kaiserslautern-Landau, Paul Ehrlich-Str.23, Building 23, 67663 Kaiserslautern, Germany.

[$]**Equal contribution and last authorship**: These authors contributed equally to this work and share last authorship

To whom correspondence should be addressed: Prof. Andrea Pautz, Department of Pharmacology, University Medical Center of the Johannes Gutenberg University, Langenbeckstraße 1, Building 708, 55131 Mainz, Germany; Tel. + 49 (6131) 17-9276; Fax, + 49 (6131) 17-9042; E-Mail: pautz@uni-mainz.de or Prof. Julia Weinmann-Menke, I. Department of Medicine, University Medical Center of the Johannes Gutenberg-University, Langenbeck Str. 1, 55131 Mainz, Germany Tel. + 49 (6131) 17-6774; Fax, + 49 (6131) 17-8471; E-Mail: julia.weinmann-menke@unimedizin-mainz.de

1. Antibodies for Immunostaining

Antibody	Clone no.	Company (Catalog#)
CD4	L3T4	BD Pharmingen, San Diego, CA, USA (553043)
CD8a	53-6.7	BD Pharmingen, San Diego, CA, USA (553027)
CD68	FA-11	Bio-Rad, formerly Serotec, Hercules, CA, USA (MCA1957)
F4/80	Cl:A3-1	Bio-Rad, formerly Serotec, Hercules, CA, USA (MCA497G)

Supplement Table 1: Antibodies for Immunostaining

2. Oligonulceotides used for qRT-PCR

Oligo	5'-primer	3'primer	Genbank Accession Nr.
col1a	TCGTCGGAGCAGACGGGAGTT	AGGGGGTTAGCGTCCGCTCA	NM000088.3
e-cad	ACACCCGGGACAACGTTTAT	GGGTCAGTATCAGCCGCTTT	NM001317186.1

gapdh	CCTCCGGGAAACTGTGG	AGTGGGGACACGGAAG	NM002046
hu MCP-1	AGCCACCTTCATTCCCCAAG	TCTCCTTGGCCACAATGGTC	NM002982.4
hu MMP3	TGGACCTGGAAATGTTTTGGC	GGCCAATTTCATGAGCAGCAA	NM002422.5
hu Pol2A	GCACCACGTCCAATGACAT	GTGCGGCTGCTTCCATAA	NM000937.5
hu S100A8	CCATGCCGTCTACAGGGATG	TATCCAACTCTTTGAACCAGACGT	NM001319196.1
hu TNF-α	CAAGCCTGTAGCCCATGTTG	GGTTGACCTTGGTCTGGTAGG	NM000594.4
mmp9	CGACGTCTTCCAGTACCGAG	GTTGGTCCCAGTGGGGATTT	NM004994.2
mu N-cadherin	TTTCAAGGTGGACGAGGACG	GCAAGTTGATTGGCGGGATG	NM007664.5
mu T-bet	ACCAGAACGCAGAGATCACTCA	CAAAGTTCTCCCGGAATCCTT	NM019507.2
mu Vimentin	TCCAGAGAGAGGAAGCCGAA	AAGGTCAAGACGTGCCAGAG	NM011701.4
mu CD4	QuantiTect® Primer Assay Mm_CD4_1_SG; Quiagen		
mu CD68	GGCAGCACAGTGGACATTC	CAATGATGAGAGGCAGCAAG	NM001291058.1
mu c-myc	ATTTCCTTTGGGCGTTGGAAAC	GGGAAGCAGCTCGAATTTCTTC	NM010849.4
mu Col 1	GCTCCTCTTAGGGGCCACT	CCACGTCTCACCATTGGGG	NM007742.4
mu Col 3	CCTTCCAGGACAACCAGGTC	CGGATAGCCACCCATTCCTC	NM009930.2
mu Col 4	ACAACCTGGGGCTAAAGGTG	TTCCTGCTCTTCCTGGCATG	NM009931.2
mu IL10	CCCTGGGTGAGAAGCTGAAG	CGGAGAGAGGTACAAACGAGG	NM010548.2
mu iNOS	CAGCTGGGCTGTACAAACCTT	CATTGGAAGTGAAGCGTTTCG	NM010927.4
mu IP10	GTCTGAGTGGGACTCAAGGGATC	CACTGGCCCGTCATCGATAT	NM021274.2
mu MMP3	TGGAGATGCTCACTTTGACG	ATGGAAACGGGACAAGTCTG	NM010809.2
mu Pol2a	ACCACGTCCAATGATATTGTGGAG	ATGTCATAGTGTCACACAGGAGCG	NM001291068.1
mu TGF-β	TGAGGTCACTGGAGTTGTACGG	GGTTCATGTCATGGATGGTGC	NM011577.2
mu TNF-α	CATCCTTCTCAAAATTCGAGTGACAA	TGGGAGTAGACAAGGTACAACCC	NM013693.3
mu αSMA	GTCCCAGACATCAGGGAGTAA	TCGGATACTTCAGCGTCAGGA	NM007392.3
n-cad	GAAGGACAGCCTCTTCTCAATGTG	TCACGGCATACACCATGCCATC	NM001792.4
pai-1	CACAAATCAGACGGCAGCACT	CATCGGGCGTGGTGAACTC	NM000602.4
snail1	ACCACTATGCCGCGCTCTT	GGTCGTAGGGCTGCTGGAA	NM005985
tgf-β	GCCCTGGACACCAACTATTG	CGTGTCCAGGCTCCAAATG	NM000660
twist1	TACGCCTTCTCGGTCTGGAG	CCCCACGCCCTGTTTCTTTG	NM000660
vim	AGGCGAGGAGAGCAGGATTT	GGGTATCAACCAGAGGGAGTGA	NM003380
zeb1	TATTCTCATTGTGGAGAGATGACTT	AGTCAGCTGCATCTGTAACACT	NM001128128

Supplement Table 2: Oligonulceotides used for qRT-PCR.

3. Supplemental Materials and Methods

3.1 Materials

All oligonucleotides were purchased from Sigma, Deisenhofen, Germany. All cell culture grade plastic materials were obtained from Greiner, Solingen, Germany. The High-Capacity cDNA Reverse Transcription Kit was purchased from Applied Biosystems, Darmstadt, Germany. The AMPLIFYME SG Universal Mix, was obtained from 7Bioscience, Neuenburg am Rhein, Germany.

3.2 Animal experiments

We purchased female C57BL/6 (B6) (6 weeks of age, 18g ±1g) mice from Charles River Laboratory. All mice were housed in accordance with standard animal care requirements. The animal studies were approved by the ethical board (23 177-07/ G 16-1-021) and were performed in accordance with the German animal protection law and the guidelines for the use of experimental animals as stipulated by the Guide of Care and Use of Laboratory Animals of the National Institutes of Health.

3.3 Ischemia/Reperfusion (I/R).

We anesthetized mice with 1.5-2.5% isoflurane being added to the respiratory air and exposed the right kidney through a flank incision. We induced unilateral ischemia of the right kidney by clamping the renal pedicle with nontraumatic microaneurysm clamps (Roboz Surgical Instrument Co.). Clamps were removed after 45 minutes. Body temperature was controlled at 36.8°C–37.2°C throughout the procedure. Oxa (1mg/kg) or dexamethasone (2.5mg/kg) application was performed every other day by intraperitoneal injection for seven days, starting immediately or after one week of I/R injury. PBS/10% EtOH was used as solvent control. Seven or 20 days after I/R injury mice were sacrificed.

3.4 Compounds

Oxacyclododecindione (Oxa) was isolated from fermentations of the imperfect fungus *Exserohilum rostratum* by chromatographic methods as previously described (1). The purity of Oxa as estimated by

HPLC-DAD/MS analysis was greater than 99 %. 14-Deoxy-14-methyloxacyclododecindione was prepared as recently described (2).

3.5 Renal histopathology

Kidney pathology was assessed as described before (3). Briefly, kidneys were fixed in 10% neutral buffered formalin for 24 h and embedded in paraffin. Stained paraffin sections (4 µm) with periodic acid-Schiff reagent, hematoxylin and eosin were assessed. At the end, the evaluated scores were summed and divided by the number of high power fields (hpf) counted and reported as the total score.

3.6 Sirius Red/Goldner Staining

The institute of pathology of the medical center of the Johannes Gutenberg University performed according to the routinely used protocols the Goldner, PAS and HE staining. Sirius Red staining was performed with 4 µm paraffin sections and picro Sirius Red in saturated aqueous solution of picric acid (Sigma, Deisenhofen, Germany). Evaluation of both stains was performed quantitatively using ImageJ (https://imagej.nih.gov/ij/index.html; version 1.53c). We analyzed the percentage of blue (Goldner staining) or red (Sirius red staining) stained parts of 10 randomly selected high-power fields (hpf) of cortex and 3 randomly selected hpf of medulla of each kidney. The percentage was averaged in each case and reported as total percentage.

3.7 Immunostainings

Kidney tissue was processed and stained for presence CD4, CD8a, CD68 and F4/80 (Supplement Table 1), as described preciously for the kidney (4).

3.8 Analysis of mRNA expression in kidney of C57BL/6 (B6) mice and human tubulus epithelial cells

To analyze the mRNA expression of different immune relevant genes in cells or mouse tissue, we prepared total RNA by homogenizing the sample in guanidiniumisothiocyanat-buffer and isolated the RNA as described. Gene expression in samples was quantified in a two-step real-time RT-PCR (qRT-PCR) as previously described (5) with the oligonucleotides listed in Supplement table 2. Specific mRNA

expression was normalized to RNA Polymerase IIa (Pol2A) or TATA Box-Binding Protein (TBP) mRNA expression. To calculate the relative mRNA expression the 2(ΔΔC(T)) method was used (6). Oligonucleotides and probes used for qRT-PCR are listed in supplement table 2.

For qRT-PCR analysis, HK2 cells were plated into six well plates at a density of 1 x 10^6 cells per well in DMEM medium overnight and starved for 24 h in DMEM medium with 0.5 % FCS. After treatment with test compound for 1 h, the cells were induced with 5 ng/mL TGF-β for 7 h. Following stimulation, the cells were lysed with 1 mL TRIzol reagent (Thermo Fisher Scientific, Waltham, USA) and total RNA was prepared according to the manufacturer's instructions. First strand cDNA was generated using the RevertAid H Minus First Strand cDNA Synthesis Kit (Fermentas, St. Leon-Roth, Germany). Gene expression was quantified from 300 ng cDNA using the 5x HOT FIREPoly® EvaGreen® qPCR Supermix (Solis Biodyne, Tartu, Estonia) according to manufacturer's suggestions with the following gene-specific primers (Supplement table 2).

Measurements were done using the StepOnePlus real-time PCR System (Thermo Fisher Scientific, Waltham, USA) with the following protocol: initial activation of HotStar Taq DNA polymerase at 95°C for 12 min, 40 cycles of 95°C for 15 s, annealing at 56°C for 30 s, extension/detection at 72°C for 30 s. Relative mRNA amounts were determined using the mathematical model for relative quantification in real-time PCR proposed by Pfaffl et al. (7).

3.9 Tissue preparation and cell culture.

Human TECs were isolated from a piece of kidney (discarded healthy tissue from nephrectomies) in the same manner as described before (8). Stimulation was performed with a cytokine mix (CM) (hu IFN-γ (300 U/mL), IL-1β (600 U/mL) and TNFα (37 ng/mL)) for 2 or 24 hours. Ethics approval number 837.467.13 and 2019-14695.

3.10 Transient Transfection and Cell Viability

The reporter plasmid (AGCCAGACA)9MLP-Luc contains nine tandem copies of the CAGA Smad binding element upstream of the adenovirus major late promoter driving luciferase expression (9). The control reporter vector pRL-EF1α for data normalization was purchased from Promega (Dual-Luciferase-Reporter-Assay). Luciferase-based reporter gene expression was thereby normalized for transfection variability and cytotoxicity against renilla expression of the constitutively active vector control (pRL-EF1α) assayed in the same sample. Transient transfections of HK2 cells were performed in 24 well cell culture plates using the transfection reagent jetPrime (Polyplus-transfection SA, Strasbourg, France) as described by the manufacturer. For induction of luciferase expression the cells were treated with 5 ng/mL TGF-β for 24 h. Luciferase activity was measured with a luminometer, using the Dual-Glo Luciferase assay system (Promega, Mannheim, Germany) according to the manufacturer´s instructions. To evaluate the effect of Oxa on cellular proliferation and viability, a Giemsa stain-based cell viability assay was performed after 48 h as previously described (10).

3.11 HK2 cell stimulation

As cellular model the human tubule epithelial cell line human kidney 2 (HK2, ATCC CRL-2190) was used. HK 2cells were seeded in petri dishes with a diameter of 10 cm with a density of 1 x 10^6 cells/mL DMEM containing 10 % FCS and grown to 70-80 % confluency. The cells were then starved in medium containing 0.5 % FCS for 24 hours, pretreated for 1 h with or without different concentrations of test compound and induced with 5 ng/mL TGF-β as indicated. Total cell extracts were obtaining by washing the cells two times in ice cold PBS and resuspending the cell pellet in 200 µl ice cold RIPA buffer (150 mM NaCl, 5 mM EDTA (pH 8.0), 50 mM Tris (pH 8.0), 1.0% V/V NP-40, 0.5% V/V sodium deoxycholate, 0.1% SDS, 5 mM sodium orthovanadate, 10 mM sodium fluoride). The protein content was determined using the PierceTM BCA Protein assay kit (Thermo Fisher Scientific, Waltham, USA).

3.12 Western Blot

For western blotting same amounts of protein (50-100 µg) were separated by 10% SDS-PAGE, transferred to nitrocellulose membranes, and subjected to immunoblotting. The membranes were probed with specific antibodies against N-cadherin (D4R1H, 13116), E-cadherin (24E10, 3195), Snail (C15D3, 3879), α-tubulin (11H10;2125) and acetyl-α-tubulin (D20G3,Lys40;5335)(all Cell Signaling Technology, Danvers, USA) Specific antibodies against β-actin (13E5; 4970, Cell Signaling Technology, Danvers, USA) served as an endogenous control. For detection appropriate secondary antibodies conjugated with horseradish peroxidase were used and signals were visualized by the enhanced chemoluminescence detection system (New England Biolabs GmbH, Frankfurt, Germany).

3.13 Zymography

For the analysis of secreted proteins, serum starved HK2 cells were pretreated with or without the compounds for 1 h and stimulated with 5 ng/ mL TGF-β. The cell culture supernatant was collected, centrifuged at 5000 x g for 10 min at 4 °C to remove cellular debris and precipitated with 10% V/V TCA at -20°C. The samples were thawed on ice, centrifuged at 12000 x g at 4 °C for 30 min and washed with 9 volumes ice cold acetone. After centrifugation at max speed and 4°C the pellet was resuspended in a buffer containing 1% W/V SDS, 60 mM Tris-HCL, pH 6.8. 25 µL of 10-fold concentrated supernatant was analyzed for activity of matrix metalloproteinases by gelatin-zymography as described (11).

3.14 Mass Spectometry

The HK2 cells were seeded in petri dishes with a diameter of 10 cm with a density of 1 x 10^6 cells/mL DMEM containing 10 % FCS and grown to 70-80 % confluency. The cells were then starved in DMEM medium without FCS for 24 hours. Then the medium was replaced, and the cells were pretreated for 1 h with or without different concentrations of test compound and induced with 5 ng/mL TGF-β for 48 h. The cell culture supernatant was collected, centrifuged at 5000 x g for 10 min at 4 °C to remove cellular debris and precipitated with 10% V/V TCA at -20°C. The samples were thawed on ice, centrifuged at 12000 x g at 4 °C for 30 min and washed with 9 volumes ice cold acetone. After centrifugation at max speed and 4°C the pellet was resuspended in 100 µL buffer containing 25 mM

ammonium bicarbonate and 8 M urea, pH 7.9. Proteins were digested and peptides desalted as described in Hamel et al. 2018 (12)(12)(4)(12)(12)(13). MS analysis was performed on a high resolution LC-MS system (Eksigent nanoLC425 coupled to a Triple-TOF 6600, AB Sciex) in information dependent acquisition (IDA) mode. The RP-HPLC separation was performed in trap-elution mode using a TriArt C18 material (TrAart C18, 5 µm particles, 0.5 x 5 mm as trapping column and TriArt C18, 3 µm particles, 300 µm x 150 mm as analytical column, YMC). A constant flow of 4 µL/min was employed and the gradient ramped within 2 min from 2% to 5% of HPLC buffer B (buffer A: 2% acetonitrile, 0.1% formic acid; buffer B: 90% acetonitrile, 0.1% formic acid), within 64 min to 35% buffer B, then within 6 min to 50% buffer B followed by washing and equilibration steps. The mass spectrometer as was run in DDA mode recording 1 survey scan (250 ms, 350-1250 m/z) and fragment spectra (50 ms, 100-1500 m/z) of the 35 most intense parent ions (charge state >2, intensity >400cps, exclusion for 6 s after one occurrence) resulting in a cycle time of 2 s. The analysis of MS runs was performed using MaxQuant version 1.6.0.16 (13). Library generation for peptide spectrum matching was based on *Homo sapiens* Proteome (14) and MaxQuant contaminants list. Oxidation of methionine, acetylation of the N-terminus and Phospho(STY) were considered as variable peptide modifications. Maximal missed cleavages were set to 3 and peptide length to 6 amino acids, the maximal mass to 6000 Da. Thresholds for peptide spectrum matching and protein identification were set by a false discovery rate (FDR) of 0.01. Relative protein expression was calculated using label free quantification (LFQ) intensities. Normalisation of protein abundance, statistical analyses and visualisation were performed with PANDA-View using the integrated SAM package (https://doi.org/10.1093/bioinformatics/bty408). Heat-maps were created with gplots implemented in R (Warnes, G. et al. 2015, Gplots: various R programming tools for plotting data. R Package. version 2.17.0. http://cran.r-project.org/package=gplots).

3.15 Statistics

Data represent means ± SEM. Statistical differences were determined by factorial analysis of variance followed by "Tukey's" or "Dunnett's" multiple comparison test. In the case of two means, classical t

test analyses were used. Two-way Anova analysis followed by Bonferroni's multiple comparisons test was performed. All statistical analyses were performed using GraphPad Prism 9.0.

4. Supplement Figure 1: Influence of OXA on the secretome of HK2 cells

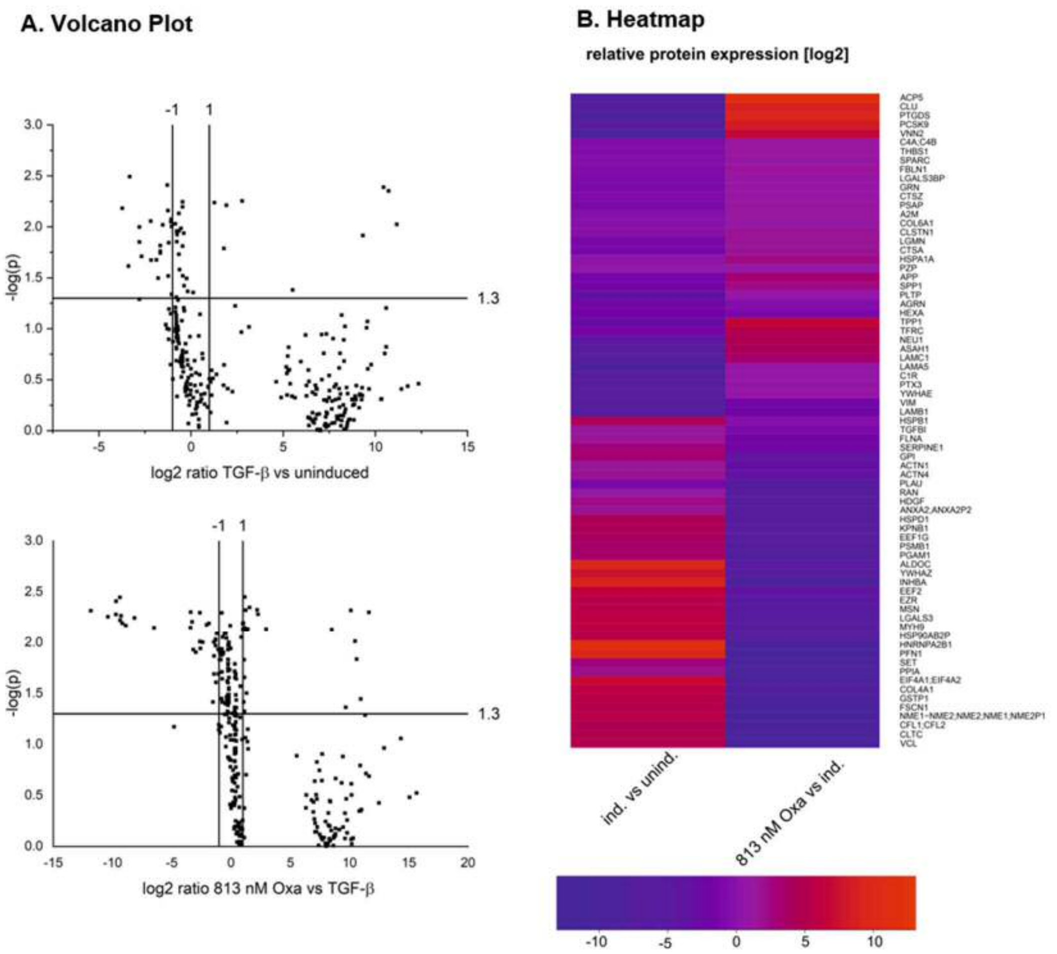

Supplement Figure 1: Influence of OXA on the secretome of HK2 cells

(A) Influence of OXA on the secretome of HK2 cells. Values are expressed as ratios (log2) of relative secreted protein levels of stimulated (48 h, 5 ng/mL TGF-β) versus un-stimulated cells as control and Oxa pre-treated (813 nM) and stimulated versus untreated, stimulated cells. Data are shown as mean values of four independent experiments ($p < 0.05$). (B) Heatmap of the significantly altered proteins (adjusted $p < 0.05$). Individual rows represent single proteins and graduated scale color codes from red (increased expression levels) to blue (decreased expression levels) Each column represents mean normalized data from n = 4 independent experiments.

5. Supplemental Reference

S1. Erkel G, Belahmer H, Serwe A, Anke T, Kunz H, Kolshorn H, et al. Oxacyclododecindione, a novel inhibitor of IL-4 signaling from Exserohilum rostratum. J Antibiot (Tokyo). 2008;61(5):285-90.

S2. Weber C, Vierengel N, Walter T, Behrendt T, Lucas T, Erkel G, et al. Total synthesis and biological evaluation of seven new anti-inflammatory oxacyclododecindione-type macrolactones. Org Biomol Chem. 2020;18(30):5906-17.
S3. Menke J, Zeller GC, Kikawada E, Means TK, Huang XR, Lan HY, et al. CXCL9, but not CXCL10, promotes CXCR3-dependent immune-mediated kidney disease. J Am Soc Nephrol. 2008;19(6):1177-89.
S4. Henke J, Erkel G, Brochhausen C, Kleinert H, Schwarting A, Menke J, et al. The fungal lactone oxacyclododecindione is a potential new therapeutic substance in the treatment of lupus-associated kidney disease. Kidney Int. 2014;86(4):780-9.
S5. Schmidtke L, Meineck M, Saurin S, Otten S, Gather F, Schrick K, et al. Knockout of the KH-Type Splicing Regulatory Protein Drives Glomerulonephritis in MRL-Fas(lpr) Mice. Cells. 2021;10(11).
S6. Livak KJ, Schmittgen TD. Analysis of relative gene expression data using real-time quantitative PCR and the 2(-Delta Delta C(T)) Method. Methods. 2001;25(4):402-8.
S7. Pfaffl MW. A new mathematical model for relative quantification in real-time RT-PCR. Nucleic Acids Res. 2001;29(9):e45.
S8. Faust J, Menke J, Kriegsmann J, Kelley VR, Mayet WJ, Galle PR, et al. Correlation of renal tubular epithelial cell-derived interleukin-18 up-regulation with disease activity in MRL-Faslpr mice with autoimmune lupus nephritis. Arthritis Rheum. 2002;46(11):3083-95.
S9. Dennler S, Itoh S, Vivien D, ten Dijke P, Huet S, Gauthier JM. Direct binding of Smad3 and Smad4 to critical TGF beta-inducible elements in the promoter of human plasminogen activator inhibitor-type 1 gene. EMBO J. 1998;17(11):3091-100.
S10. Mirabelli CK, Bartus H, Bartus JO, Johnson R, Mong SM, Sung CP, et al. Application of a tissue culture microtiter test for the detection of cytotoxic agents from natural products. J Antibiot (Tokyo). 1985;38(6):758-66.
S11. Kupai K, Szucs G, Cseh S, Hajdu I, Csonka C, Csont T, et al. Matrix metalloproteinase activity assays: Importance of zymography. J Pharmacol Toxicol Methods. 2010;61(2):205-9.
S12. Sommer F, Muhlhaus T, Hemme D, Veyel D, Schroda M. Identification and Validation of Protein-Protein Interactions by Combining Co-immunoprecipitation, Antigen Competition, and Stable Isotope Labeling. Methods Mol Biol. 2014;1188:245-61.
S13. Cox J, Mann M. MaxQuant enables high peptide identification rates, individualized p.p.b.-range mass accuracies and proteome-wide protein quantification. Nat Biotechnol. 2008;26(12):1367-72.
S14. Merchant SS, Prochnik SE, Vallon O, Harris EH, Karpowicz SJ, Witman GB, et al. The *Chlamydomonas* genome reveals the evolution of key animal and plant functions. Science. 2007;318(5848):245-50.

6: Supplemental Secretome_TGF_Oxa_log2Ratio

overview of significantly Oxa-regulated proteins in cell culture supernatant of TGF-β induced HK2 cells.

protein name	mean value (log2) pos. vs neg.	p-value	mean value (log2) Oxa treated vs pos.	p-value	molecular function	cellular component
A2M	-0,23	n.s.	1,13	0,0072	protease binding; serine-type endopeptidase inhibitor activity; signaling receptor binding; protein binding; growth factor binding; enzyme binding; interleukin-8 binding; interleukin-1 binding; tumor necrosis factor binding; calciumdependent protein binding	extracellular exosome; blood microparticle
ACP5	-7,33	0,014	10,07	0,0048	ferrous iron binding; ferric iron binding	extracellular exosome
ACTN1	1,4	n.s.	-2,74	0,0051	double-stranded RNA binding; integrin binding; integrin binding; protein binding; vinculin binding; vinculin binding; liganddependent nuclear receptor transcription coactivator activity; protein homodimerization activity; ion channel binding; actin filament binding	stress fiber; ruffle; extracellular space; intracellular; cytoplasm; cell-cell junction; focal adhesion; focal adhesion; cell projection; extracellular exosome; actin filament
ACTN4	1,61	n.s.	-3,3	0,0063	RNA polymerase II regulatory region sequence-specific DNA binding; nucleoside binding; RNA binding; protein binding; ligand-dependent nuclear receptor transcription coactivator activity; chromatin DNA binding; nuclear hormone receptor binding; protein homodimerization activity; retinoic acid receptor binding; ion channel binding; actin filament binding; actin filament binding	extracellular space; intracellular; nucleus; cytoplasm; cytosol; focal adhesion; nuclear body; intracellular ribonucleoprotein complex; perinuclear region of cytoplasm; extracellular exosome; actin cytoskeleton
AGRN	-1,33	0,007	-0,38	0,0371	protein binding	basal lamina; cytosol; plasma membrane; extracellular matrix; extracellular exosome
ALDOC	8,55	0,009	-4,96	n.s.	fructose-bisphosphate aldolase activity; fructose-bisphosphate; protein binding; cytoskeletal protein binding	extracellular exosome
ANXA2;AN XA2 P2	1,68	n.s.	-5,95	0,0108	protease binding; RNA binding; protein binding; calcium-dependent phospholipid binding; phosphatidylinositol-4,5bisphosphate binding; phospholipase A2 inhibitor activity; identical protein binding; S100 protein binding; calcium-dependent protein binding; cadherin binding involved in cell-cell adhesion; molecular function regulator	extracellular region; extracellular space; nucleus; cytoplasm; lysosomal membrane; endosome; lipid droplet; plasma membrane; cell-cell adherens junction; cell surface; membrane; midbody; extracellular matrix; late endosome membrane; vesicle; extracellular exosome; PCSK9-AnxA2 complex; membrane raft
APP	-1,36	0,03	2,87	0,0074	serine-type endopeptidase inhibitor activity; signaling receptor binding; protein binding; enzyme binding; identical protein binding; PTB domain binding	extracellular space; nuclear envelope lumen; cytoplasm; endosome; Golgi apparatus; plasma membrane; cell surface; dendritic spine; dendritic shaft; receptor complex; membrane raft; synapse; perinuclear region of cytoplasm; extracellular exosome
ASAH1	-5,86	0,009	3,67	n.s.	member of the acid ceramidase family of proteins	extracellular space; extracellular exosome
C1R	-5,03	0,015	0,78	0,027	serine-type endopeptidase activity; calcium ion binding; protein binding	extracellular space; extracellular exosome; blood microparticle
C4A	-0,52	0,03	1,15	0,0036	complement component C1q binding	extracellular space; axon; dendrite; neuronal cell body; synapse; extracellular exosome; blood microparticle
C4B	-0,52	0,03	1,15	0,0036	complement binding; carbohydrate binding	extracellular space; other organism cell; extracellular exosome; blood microparticle
CFL1	4,27	n.s.	-9,21	0,0068	protein binding; actin filament binding	extracellular space; cytoplasm; focal adhesion; membrane; extracellular matrix; vesicle; extracellular exosome
CFL2	4,27	n.s.	-9,21	0,0068	protein binding	extracellular space; Z disc; I band; extracellular exosome
CLSTN1	-0,12	n.s.	1,34	0,0074	amyloid-beta binding; protein binding; kinesin binding; X11-like protein binding	extracellular exosome

CLTC	4,66	n.s.	-9,39	0,0065	RNA binding; double-stranded RNA binding; protein binding; clathrin light chain binding; low-density lipoprotein particle receptor binding; disordered domain specific binding; ubiquitin-specific protease binding	lysosome; endosome; spindle; focal adhesion; membrane; clathrin coat; clathrin-coated vesicle; extracellular matrix; extracellular exosome; clathrin complex; extracellular vesicle; mitotic spindle, microtubule
CLU	-6,94	0,003	8,28	0,0051	amyloid-beta binding; protein binding; ubiquitin protein ligase binding; lowdensity lipoprotein particle receptor binding; misfolded protein binding; misfolded protein binding; ATPase activity	extracellular region; extracellular space; cytoplasm; mitochondrion; cell surface; extracellular matrix; spherical high-density lipoprotein particle; perinuclear region of cytoplasm; extracellular exosome; blood microparticle; neurofibrillary tangle; apical dendrite; cell periphery
COL4A1	5,35	n.s.	-7,54	0,01	extracellular matrix structural constituent; protein binding; platelet-derived growth factor binding	collagen type IV trimer; extracellular matrix
COL6A1	-0,09	n.s.	0,98	0,0065	platelet-derived growth factor binding	extracellular region; lysosomal membrane; membrane; extracellular matrix; extracellular exosome
CTSA	-1,09	0,01	1,33	0,0075	serine-type carboxypeptidase activity	nucleoplasm; membrane; intracellular membranebounded organelle; extracellular exosome
CTSZ	-0,67	0,019	1,04	0,0064	carboxypeptidase activity; protein binding	extracellular space; lysosome; endoplasmic reticulum; cytoplasmic vesicle; intracellular membrane-bounded organelle; extracellular exosome; cell cortex region
EEF1G	3,53	n.s.	-5,62	0,0067	protein binding; cadherin binding	nucleus; cytoplasm; membrane; extracellular exosome; endoplasmic reticulum
EEF2	5,67	n.s.	-3,34	0,0248	RNA binding; protein binding; protein kinase binding; cadherin binding	nucleus; cytoplasm; cytosol; plasma membrane; membrane; aggresome; intracellular ribonucleoprotein complex; extracellular matrix; extracellular exosome
EIF4A1	6,53	n.s.	-8,34	0,0057	RNA binding; double-stranded RNA binding; protein binding	cytoplasm; membrane; extracellular matrix; extracellular exosome; nucleus
EIF4A2	6,53	n.s.	-8,34	0,0057	RNA binding; protein binding; ATPase activity	perinuclear region of cytoplasm
EZR	5,08	n.s.	-4,44	0,0098	RNA binding; actin binding; protein binding; microtubule binding; protein domain specific binding; protein kinase A catalytic subunit binding; protein kinase A regulatory subunit binding; S100 protein binding; cadherin binding; cell adhesion molecule binding; actin filament binding; protein kinase A binding; ATPase binding	fibrillar center; ruffle; immunological synapse; extracellular space; cytoplasm; endosome; cytosol; actin filament; plasma membrane; microvillus; focal adhesion; actin cytoskeleton; membrane; apical plasma membrane; extrinsic component of membrane; filopodium; vesicle; cell projection; plasma membrane raft; apical part of cell; perinuclear region of cytoplasm; extracellular exosome; invadopodium; cell periphery; cytoplasmic side of apical plasma membrane; TCR signalosome
FBLN1	-0,48	0,012	1,31	0,0316	fibronectin binding; extracellular matrix structural constituent; calcium ion binding; protein C-terminus binding; identical protein binding; fibrinogen binding	extracellular region; proteinaceous extracellular matrix; extracellular space; extracellular exosome; elastic fiber; fibrinogen complex; extracellular matrix
FLNA	1,42	0,016	-1,7	0,0116	G-protein coupled receptor binding; RNA binding; signal transducer activity; protein binding; transcription factor binding; potassium channel regulator activity; Rho GTPase binding; Ral GTPase binding; kinase binding; small GTPase binding; Fcgamma receptor I complex binding; protein homodimerization activity; ion channel binding; cadherin binding; Rac GTPase binding; actin filament binding;	nucleus; nucleolus; cytoplasm; cytosol; plasma membrane; cell-cell junction; focal adhesion; actin cytoskeleton; membrane; extracellular matrix; Myb complex; extracellular exosome; filamentous actin

FSCN1	4,92	n.s.	-9,4	0,0036	RNA binding; actin binding; protein binding; drug binding; cadherin binding; actin filament binding	stress fiber; ruffle; podosome; cytoplasm; cytosol; cytoskeleton; microvillus; cell-cell junction; actin cytoskeleton; filopodium; cell projection membrane; microspike; extracellular exosome; invadopodium
GPI	2,8	n.s.	-3,38	0,0204	ubiquitin protein ligase binding	nucleoplasm; cytosol; plasma membrane; membrane; extracellular exosome
GRN	-0,77	0,011	1,05	0,0221	RNA binding; protein binding	lysosome; endosome; endoplasmic reticulum; extracellular exosome
GSTP1	5,01	n.s.	-7,98	0,0071	glutathione transferase activity; glutathione peroxidase activity; protein binding; S-nitrosoglutathione binding; dinitrosyl-iron complex binding	extracellular space; intracellular; mitochondrion; cytosol; vesicle; extracellular exosome; TRAF2-GSTP1 complex
HDGF	2,17	n.s.	-6,38	0,0095	RNA polymerase II transcription corepressor activity; transcription corepressor binding; RNA binding	extracellular region; extracellular space; nucleoplasm; transcriptional repressor complex; extracellular matrix
HEXA	-1,67	0,01	-0,95	n.s.	protein binding; acetylglucosaminyltransferase activity; protein heterodimerization activity	membrane; azurophil granule; extracellular exosome
HNRNPA2B1	10,27	0,004	-9,75	0,0039	RNA binding; mRNA 3'-UTR binding; protein binding; miRNA binding; singlestranded telomeric DNA binding; N6methyladenosine-containing RNA binding	nucleus; nucleoplasm; spliceosomal complex; cytoplasm; membrane; intracellular ribonucleoprotein complex; extracellular exosome; catalytic step 2 spliceosome
HSP90AB2P	5,11	n.s.	-5,91	0,0118	Heat Shock Protein 90 Alpha Family Class B Member 2, Pseudogene	extracellular exosome
HSPA1A	0,16	n.s.	2,16	0,0053	RNA polymerase II transcription corepressor activity; G-protein coupled receptor binding; RNA binding; signaling receptor binding; protein binding; ATP binding; ATPase activity; enzyme binding; heat shock protein binding; denatured protein binding; ubiquitin protein ligase binding; ATPase activity, coupled; histone deacetylase binding; protein binding involved in protein folding; cadherin binding; protein N-terminus binding; unfolded protein binding; C3HC4-type RING finger domain binding; disordered domain specific binding	nucleus; cytoplasm; centrosome; centriole; cytosol; focal adhesion; inclusion body; aggresome; nuclear speck; intracellular ribonucleoprotein complex; vesicle; perinuclear region of cytoplasm; extracellular exosome; blood microparticle; ubiquitin ligase complex
HSPB1	4,15	0,012	-0,29	0,0186	RNA binding; protein binding; protein kinase binding; identical protein binding; identical protein binding; protein homodimerization activity; protein binding involved in protein folding	extracellular space; nucleus; cytoplasm; focal adhesion; extracellular matrix; extracellular exosome
HSPD1	4,07	n.s.	-6,21	0,0082	lipopolysaccharide binding; p53 binding; RNA binding; double-stranded RNA binding; protein binding; high-density lipoprotein particle binding; enzyme binding; ubiquitin protein ligase binding; apolipoprotein binding; apolipoprotein A-I binding; chaperone binding	extracellular space; cytoplasm; mitochondrion; mitochondrial matrix; early endosome; cytosol; plasma membrane; clathrin-coated pit; cell surface; membrane; coated vesicle; extracellular matrix; lipopolysaccharide receptor complex; extracellular exosome; cyclindependent protein kinase activating kinase holoenzyme complex
INHBA	8,27	n.s.	-7,75	0,0118	cytokine activity; protein binding; peptide hormone binding; identical protein binding; type II activin receptor binding	extracellular region; activin A complex; inhibin A complex
KPNB1	3,92	n.s.	-5,53	0,0094	RNA binding; protein binding; enzyme binding; protein domain specific binding	nuclear envelope; nucleoplasm; cytosol; membrane; nuclear membrane; extracellular exosome; endoplasmic reticulum tubular network
LAMA5	-8,1	0,007	0,88	n.s.	integrin binding	basement membrane; extracellular space; nucleus; extracellular matrix; laminin-10 complex; extracellular exosome
LAMB1	-5,72	0,032	-2,02	n.s.	extracellular matrix structural constituent; protein binding	basement membrane; laminin-1 complex; laminin-1 complex; laminin-2 complex; extracellular space; extracellular matrix; laminin-8 complex; laminin-10

							complex; extracellular exosome
LAMC1	-5,54	0,021	3,3	n.s.	extracellular matrix structural constituent	basement membrane; extracellular matrix; extracellular exosome
LGALS3	5,48	n.s.	-5,41	0,01	RNA binding; protein binding; IgE binding; chemoattractant activity; laminin binding	immunological synapse; extracellular region; extracellular space; nucleus; cytoplasm; mitochondrial inner membrane; membrane; extracellular matrix; extracellular exosome
LGALS3BP	-0,76	n.s.	1,08	0,0148	no Info	extracellular region; extracellular space; extracellular matrix; extracellular exosome; blood microparticle
LGMN	-1,11	0,008	1,53	0,0045	peptidase activity	extracellular exosome
MSN	5,14	n.s.	-4,9	0,0125	double-stranded RNA binding; signaling receptor binding; protein binding; enzyme binding; protein kinase binding; cell adhesion molecule binding	extracellular space; nucleus; cytoplasm; cytosol; plasma membrane; microvillus; focal adhesion; cell surface; apical plasma membrane; filopodium; pseudopodium; vesicle; apical part of cell; perinuclear region of cytoplasm; extracellular exosome; invadopodium; cell periphery; blood microparticle
MYH9	5,43	n.s.	-5,44	0,0205	microfilament motor activity; RNA binding; actin binding; protein binding; ATP binding; ATPase activity; protein domain specific binding; actin-dependent ATPase activity; protein homodimerization activity; protein membrane anchor; ADP binding; cadherin binding; actin filament binding	stress fiber; ruffle; uropod; nucleus; cytoplasm; actomyosin contractile ring; cytosol; plasma membrane; actin cytoskeleton; membrane; myosin II complex; extracellular matrix; cell leading edge; cleavage furrow; actomyosin; extracellular exosome; myosin II filament; immunological synapse; COP9 signalosome; integrin complex
NEU1	-4,07	0,021	4,31	0,0147	exo-alpha-sialidase activity; protein binding; alpha-sialidase activity	lysosome; cell junction; intracellular membrane-bounded organelle; extracellular exosome
NME1	5,13	n.s.	-9,85	0,0053	magnesium ion binding; RNA binding; deoxyribonuclease activity; nucleoside diphosphate kinase activity; nucleoside diphosphate kinase activity; protein binding; ATP binding; GTP binding; identical protein binding; ribosomal small subunit binding	nucleus; cytosol; membrane; ruffle membrane; extracellular exosome
NME1-NME2	5,13	n.s.	-9,85	0,0053	no Info	no Info
NME2	5,13	n.s.	-9,85	0,0053	nucleoside diphosphate kinase activity; nucleoside diphosphate kinase activity; protein binding	ruffle; nucleus; cytoplasm; lamellipodium; extracellular exosome; cell periphery; focal adhesion
NME2P1	5,13	n.s.	-9,85	0,0053	no Info	nucleus; extracellular exosome
PCSK9	-6,13	0,024	7,44	0,0098	RNA binding; serine-type endopeptidase activity; protein binding; sodium channel inhibitor activity; receptor inhibitor activity; apolipoprotein receptor binding; protein self-association; low-density lipoprotein particle receptor binding; low-density lipoprotein particle receptor binding; verylow-density lipoprotein particle receptor binding	extracellular space; cytoplasm; lysosome; early endosome; late endosome; endoplasmic reticulum; Golgi apparatus; plasma membrane; cell surface; perinuclear region of cytoplasm; PCSK9-LDLR complex; PCSK9AnxA2 complex
PFN1	8,81	0,004	-10,58	0,0056	adenyl-nucleotide exchange factor activity; RNA binding; actin binding; actin monomer binding; protein binding; phosphatidylinositol-4,5-bisphosphate binding; cadherin binding; proline-rich region binding	nucleus; cytoplasm; focal adhesion; cell cortex; membrane; extracellular exosome; blood microparticle
PGAM1	3,14	n.s.	-7,19	0,005	phosphoglycerate mutase activity; phosphoglycerate mutase activity; phosphoglycerate mutase activity; protein binding; protein kinase binding	cytoplasm; cytosol; membrane; extracellular exosome
PLAU	-1,11	0,009	-5,75	0,0136	protein binding	extracellular space; focal adhesion; cell surface; extracellular exosome
						lipid transporter activity; phospholipid transporter activity;	

PLTP	-3,02	0,046	0,65	n.s.	phosphatidylethanolamine binding; phosphatidylcholine transporter activity; diacylglycerol binding; phosphatidylcholine binding; ceramide transporter activity; phosphatidic acid binding; ceramide binding; phosphatidylglycerol binding; phosphatidylethanolamine transporter activity; phosphatidic acid transporter activity	extracellular space; high-density lipoprotein particle
PPIA	1,86	0,006	-12	0,0049	RNA binding; peptidyl-prolyl cis-trans isomerase activity; protein binding; cyclosporin A binding	extracellular space; nucleus; cytosol; focal adhesion; membrane; vesicle; extracellular exosome
PSAP	-0,87	0,014	1,22	0,0048	beta-galactosidase activity; protein binding; phospholipid binding; protein homodimerization activity; ganglioside GM1 binding; ganglioside GM2 binding; ganglioside GM3 binding; ganglioside GT1b binding; ganglioside GP1c binding	extracellular region; extracellular space; lysosome; extracellular matrix; intracellular membrane-bounded organelle; extracellular exosome
PSMB1	3,3	n.s.	-6,65	0,0072	protein binding	proteasome complex; nucleus; extracellular exosome
PTGDS	-7,79	0,01	8,68	0,0359	prostaglandin-D synthase activity; fatty acid binding; protein binding	extracellular region; extracellular space; rough endoplasmic reticulum; extracellular exosome
PTX3	-5,29	0,017	0,78	0,027	complement component C1q binding; protein binding; identical protein binding; virion binding	extracellular space
PZP	0,64	n.s.	0,99	0,0074	noInfo	extracellular exosome; blood microparticle
RAN	1,05	n.s.	-5,68	0,0115	magnesium ion binding; RNA binding; protein binding; GTP binding; GTP binding; GDP binding; GDP binding; cadherin binding; protein heterodimerization activity; pre-miRNA binding	nucleus; nuclear envelope; nucleoplasm; nucleolus; cytoplasm; centriole; membrane; midbody; extracellular matrix; RNA nuclear export complex; recycling endosome; extracellular exosome; Flemming body;
SERPINE1	2,76	0,006	-1,97	0,0065	protease binding; serine-type endopeptidase inhibitor activity; signaling receptor binding; protein binding	extracellular region; extracellular space; extracellular matrix; extracellular exosome
SET	2,55	n.s.	-9,55	0,006	protein binding	nucleus; nucleoplasm; cytoplasm; endoplasmic reticulum; lipid droplet; perinuclear region of cytoplasm
SPARC	-0,41	n.s.	1,07	0,0386	calcium ion binding; protein binding; collagen binding	extracellular region; cytoplasm; cell surface; nuclear matrix; platelet alpha granule; platelet alpha granule membrane; mitochondrion; plasma membrane
SPP1	-1,35	0,004	2,14	0,0048	protein binding	extracellular space; Golgi apparatus; extracellular exosome
TFRC	-1,35	0,042	4,55	0,0075	RNA binding; double-stranded RNA binding; protein binding; transferrin transmembrane transporter activity; identical protein binding; protein homodimerization activity	extracellular region; extracellular space; endosome; integral component of plasma membrane; clathrincoated pit; external side of plasma membrane; cell surface; endosome membrane; basolateral plasma membrane; cytoplasmic vesicle; perinuclear region of cytoplasm; recycling endosome; extracellular exosome; blood microparticle; extracellular vesicle; HFE-transferrin receptor complex
TGFBI	1,25	0,006	-0,82	0,0051	protein binding; collagen binding	extracellular region; extracellular space; trans-Golgi network; extracellular matrix; extracellular exosome
THBS1	-0,49	n.s.	1,12	0,0476	phosphatidylserine binding; fibronectin binding; integrin binding; protein binding; heparin binding; fibroblast growth factor binding; low-density lipoprotein particle binding; laminin binding; fibrinogen binding; collagen V binding	extracellular region; fibrinogen complex; extracellular space; external side of plasma membrane; cell surface; secretory granule; extracellular matrix; platelet alpha granule; extracellular exosome
TPP1	-2,65	n.s.	5,94	0,0435	endopeptidase activity; endopeptidase activity; protein binding; peptidase activity; serine-type peptidase activity; tripeptidylpeptidase activity; tripeptidyl-peptidase activity	lysosome; lysosome; extracellular exosome

VCL	4,49	n.s.	-9,39	0,0055	dystroglycan binding; actin binding; protein binding; ubiquitin protein ligase binding; alpha-catenin binding; cadherin binding	cell-cell adherens junction; focal adhesion; costamere; extracellular exosome; extracellular vesicle
VIM	-5,77	0,014	-1,63	n.s.	double-stranded RNA binding; structural constituent of cytoskeleton; protein binding; protein C-terminus binding; protein domain specific binding; identical protein binding; scaffold protein binding; keratin filament binding	cytoplasm; peroxisome; cytosol; polysome; cytoskeleton; intermediate filament; focal adhesion; intracellular ribonucleoprotein complex; extracellular matrix; extracellular exosome; plasma membrane
VNN2	-8,45	0,02	6,25	n.s.	pantetheine hydrolase activity	no Info
YWHAE	-5,38	0,018	0,78	0,027	RNA binding; calcium channel regulator activity; protein binding; potassium channel regulator activity; enzyme binding; MHC class II protein complex binding; ubiquitin protein ligase binding; identical protein binding; histone deacetylase binding; ion channel binding; cadherin binding; protein heterodimerization activity; phosphoserine residue binding; phosphoprotein binding	nucleus; cytoplasm; focal adhesion; membrane; extracellular exosome; plasma membrane
YWHAZ	6,92	n.s.	-6,25	0,0386	RNA binding; protein binding; transcription factor binding; protein kinase binding; ubiquitin protein ligase binding; identical protein binding; ion channel binding; cadherin binding	extracellular space; nucleus; focal adhesion; vesicle; extracellular exosome; blood microparticle
